# The CCR4-NOT Complex Mediates Deadenylation and Degradation of Stem Cell mRNAs and Promotes Planarian Stem Cell Differentiation

**DOI:** 10.1371/journal.pgen.1004003

**Published:** 2013-12-19

**Authors:** Jordi Solana, Chiara Gamberi, Yuliana Mihaylova, Stefanie Grosswendt, Chen Chen, Paul Lasko, Nikolaus Rajewsky, A. Aziz Aboobaker

**Affiliations:** 1 Centre for Genetics and Genomics, University of Nottingham, Queen's Medical Centre, Nottingham, United Kingdom; 2 Systems Biology of Gene Regulatory Elements, Max-Delbrück-Center for Molecular Medicine, Berlin, Germany; 3 Department of Biology, McGill University, Montréal, Québec, Canada; 4 Department of Biology, Concordia University, Montreal, Québec, Canada; 5 Department of Zoology, University of Oxford, Oxford, United Kingdom; HHMI/University of Illinois at Urbana-Champaign, United States of America

## Abstract

Post-transcriptional regulatory mechanisms are of fundamental importance to form robust genetic networks, but their roles in stem cell pluripotency remain poorly understood. Here, we use freshwater planarians as a model system to investigate this and uncover a role for CCR4-NOT mediated deadenylation of mRNAs in stem cell differentiation. Planarian adult stem cells, the so-called neoblasts, drive the almost unlimited regenerative capabilities of planarians and allow their ongoing homeostatic tissue turnover. While many genes have been demonstrated to be required for these processes, currently almost no mechanistic insight is available into their regulation. We show that knockdown of planarian Not1, the CCR4-NOT deadenylating complex scaffolding subunit, abrogates regeneration and normal homeostasis. This abrogation is primarily due to severe impairment of their differentiation potential. We describe a stem cell specific increase in the mRNA levels of key neoblast genes after *Smed-not1* knock down, consistent with a role of the CCR4-NOT complex in degradation of neoblast mRNAs upon the onset of differentiation. We also observe a stem cell specific increase in the frequency of longer poly(A) tails in these same mRNAs, showing that stem cells after *Smed-not1* knock down fail to differentiate as they accumulate populations of transcripts with longer poly(A) tails. As other transcripts are unaffected our data hint at a targeted regulation of these key stem cell mRNAs by post-transcriptional regulators such as RNA-binding proteins or microRNAs. Together, our results show that the CCR4-NOT complex is crucial for stem cell differentiation and controls stem cell-specific degradation of mRNAs, thus providing clear mechanistic insight into this aspect of neoblast biology.

## Author Summary

Although transcriptional regulation in stem cells is a very active subject, much less is known about how post-transcriptional mechanisms of gene expression affect stem cells. Here, we use freshwater planarians in order to address this question. Planarians have a striking regenerative capacity driven by a population of pluripotent stem cells, the neoblasts. Control of both proliferation and differentiation is thought to rely heavily on post-transcriptional mechanisms, but their precise role is unknown. Poly(A) tail length regulation is an important mechanism of post-transcriptional control of gene expression as changes can be very rapid, and longer poly(A) tails are linked to increased mRNA stability and translational activity. We investigated the role of the CCR4-NOT complex, the major deadenylating complex in eukaryotes, by knocking down its main scaffolding subunit called Not1. Neoblasts in knock down animals are unable to differentiate and accumulate mRNAs with longer poly(A) tails. Our results show that the CCR4-NOT complex is needed for the targeted degradation of specific mRNAs expressed in stem cells, and the failure of this process likely prevents neoblasts from differentiating. These results reveal a new functional aspect of the CCR4-NOT complex and offer a mechanistic insight into the regulation of planarian stem cells.

## Introduction

Post-transcriptional control is central for the regulation of gene expression in stem cells [1]. A key post-transcriptional process is mRNA degradation [2]–[4] the control of which is believed to be as important as transcriptional regulation [5], [6]. Although transcriptional regulation has been extensively studied, less is known about the developmental and physiological roles of mRNA degradation in stem cells, which are thought to involve the same RNA binding proteins [7] that act together to coordinate many complex aspects of mRNA biology, one of which is degradation.

mRNA degradation starts with deadenylation (i.e. shortening of the poly(A) tail) [2], [8]. This affects gene expression both by decreasing translational activity and mRNA stability [9], [10]. The major deadenylase in eukaryotes is the CCR4-NOT complex [11]–[13], which is also involved in regulating several other aspects of mRNA metabolism, such as mRNA export [14], [15], translation [15] and transcription itself [11], [16]–[18].

In yeast, the CCR4-NOT complex is composed of nine different subunits [11]: two deadenylases (Ccr4p and Pop2p/Caf1p), five Not proteins (Not1p–Not5p), Caf40p and Caf130p. Among them, Not1p, a 240 kDa protein, is thought to act as a scaffold and is the only subunit required for yeast viability [11], [19]. Most of the subunits of the yeast complex are conserved across metazoans [20]–[22], with the exception of Not5p and Caf130p. In mammals two paralogous genes with mutually exclusive expression patterns encode each deadenylase of the complex [23]. Furthermore, the two deadenylase subunits Caf1 and Ccr4 regulate distinct sets of mRNAs [24], [25].

A number of translational repressors interact with the CCR4-NOT complex to repress their targets. For instance, Nanos proteins [26], [27], PUF proteins [28], [29], Smaug [30], and Bicaudal-C [31] all repress their target mRNAs via interaction with different subunits of the CCR4-NOT complex. Furthermore, the CCR4-NOT complex mediates the deadenylation of miRNA-targeted and piRNA-targeted mRNAs, executing the repressive functions of some small RNAs [32]–[35]. The CCR4-NOT complex directly binds to GW182, a component of miRNA repression complexes through evolutionary conserved motifs [36], [37].

Little is known, however, about the role of the CCR4-NOT complex in stem cells. It was found, for instance, that different components are important in maintaining mouse and human ESC identity [38], but the mechanisms remain largely unexplored. The freshwater planarian *Schmidtea mediterranea* is an emerging model for stem cell biology [39]–[41]. Its striking regeneration capacities are sustained by the presence of the neoblasts, a population of pluripotent stem cells that not only drive regeneration but sustain constant homeostatic cell turnover as well [42]. Planarian neoblasts can be eliminated by irradiation and are amenable to RNAi-mediated gene knock down. Furthermore, their abundance in the organism allows the quantitative evaluation of phenotypes.

The regulation of neoblasts and their pluripotency involves both transcriptional and post-transcriptional regulation, and a number of putative post-transcriptional regulators have been described to affect neoblast function [43]–[46]. Neoblasts also contain chromatoid bodies, RNA rich granules similar to germ granules which are thought to constitute hubs for mRNA processing and post-transcriptional regulation [39], [45], [47]–[49]. Recently, transcriptomic profiles of neoblasts have become available [50]–[54], confirming their long known resemblance to germ line stem cells [55], but also highlighting the importance of post-transcriptional mechanisms for their regulation [50] and the conservation of pluripotency determinants between planarian and mammalian stem cells [51], [52], [56], [57].

Here, we use *S. mediterranea* to investigate the role of the CCR4-NOT complex in stem cell regulation by characterizing the function of the *Smed-not1* gene, which encodes for the homologue of Not1p in yeast and CNot1 in mammals. We report that its knock down specifically affects the differentiation and self-maintenance capabilities of neoblasts. *Smed-not1* knock down results in a progressive increase in the levels of several neoblast transcripts, and we demonstrate that this increase is stem cell specific. Finally, we observe that these same mRNAs have stem cell specific increases in the frequency of long poly(A) tails after *Smed-not1* knock down, showing that the observed increases in mRNA levels in stem cells are likely a consequence of decreased targeted deadenylation by the CCR4-NOT complex. Our findings highlight a likely central role for poly(A) tail length regulation in orchestrating pluripotent stem cell differentiation.

## Results

### *In silico* characterization of the CCR4-NOT complex in *S. mediterranea*

We identified the different subunits of the CCR4-NOT complex in the planarian species *S. mediterranea* by TBLASTN searches in the *S. mediterranea* genome and in our reference transcriptome assembly [50], [53] and other genomic and transcriptomic resources [58]–[60] (Table S1). We identified homologues of all metazoan genes known to encode subunits of the CCR4-NOT complex. Two components of the CCR4-NOT complex, the deadenylases Ccr4 and Caf1 were previously described in the planarian species *Dugesia japonica* [45]. However, no phenotype was reported for these enzymatic components after RNAi-mediated knock down and we also observed no strong phenotype (Figure S1) for *Smed-not6* (the Ccr4 orthologue, Table S1), *Smed-not7A* and *Smed-not7B* (the two paralogues of Caf1 in *S. mediterranea*, Table S1). We instead chose to focus on *Smed-not1*, as Not1 is believed to act as the central scaffolding protein in the complex and it is the only component of the complex essential for viability in yeast [11], [19].

### *Smed-not1* is expressed in CNS and throughout the parenchyma in an irradiation-sensitive manner

**Figure 1:**
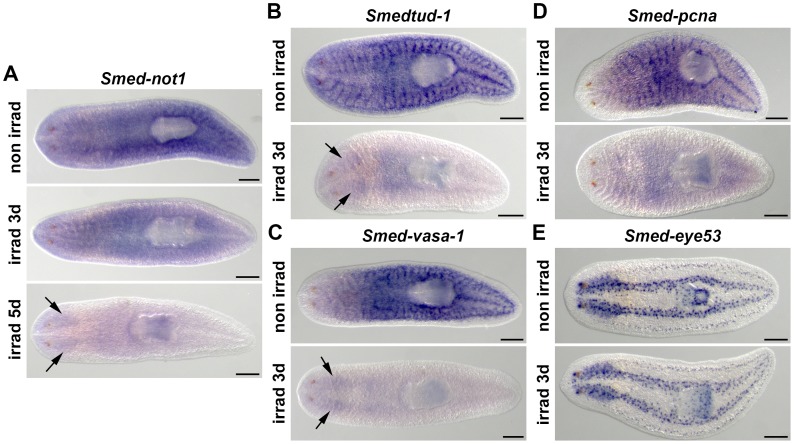
*Smed-not1* is expressed in CNS and throughout the parenchyma in an irradiation-sensitive manner. (A–D) WMISH of *Smed-not1* (A), *Smedtud-1* (B), *Smed-vasa-1* (C), *Smed-pcna* (D) and the control marker *Smed-eye53* (E), in non-irradiated and irradiated animals. *Smed-not1* signals are detected as a broad staining pattern in non-irradiated animals (A, top panel, non irrad), and decrease 3 days (A, middle panel, irrad 3d) and 5 days (A, bottom panel, irrad 5d) after lethal irradiation. *Smed-not1* signal is detected in the CNS (arrows). The neoblast specific signals of other mRNAs expressed in neoblasts disappear by day 3 of irradiation (B, C, D, irrad 3d), while signals are still detectable in the CNS for *Smedtud-1* (B, arrows), and *Smed-vasa-1* (C, arrows). No *Smed-pcna* signal is detected in the CNS (D). The differentiated cell control marker *Smed-eye53* shows no differences upon irradiation (E). Anterior is to the left. Scale bars: 500 µm.

We investigated the expression pattern of *Smed-not1* by whole mount in situ hybridization (WMISH). We observed broad expression of this key component of the core deadenylation complex, including expression throughout the parenchyma and the central nervous system (CNS) (Figure 1A, top panel). This pattern suggested to us that *Smed-not1* may be expressed in neoblasts, since they are distributed throughout the parenchyma. To test this we monitored parenchymal expression after irradiation to remove neoblasts and observed that the parenchymal component of expression disappeared over a period of 5 days after irradiation (Figure 1A, middle and bottom panels). All neoblasts disappear 24–48 hours after lethal irradiation, and consequently the expression of neoblast specific genes disappears over a similar period. As controls we analyzed the expression of *Smedtud-1*, the orthologue of the previously described *Schmidtea polychroa* Tudor gene *Spoltud-1* [48] (Figure 1B), *Smed-vasa-1*, a Vasa orthologue of *S. mediterranea* [54], [61] (Figure 1C), and *Smed-pcna*, the orthologue of the PCNA gene described in *Dugesia japonica* [62] (Figure 1D). As expected, the neoblast-specific signals of all three disappeared by day 3 post-irradiation, while irradiation insensitive expression in differentiated cells of the CNS remained for *Smedtud-1* and *Smed-vasa-1* (Figure 1B–D). *Smed-eye53*, a marker expressed in differentiated cells [63], was used as a control to demonstrate that gene expression in post-mitotic cells is not ablated by irradiation (Figure 1E). Since *Smed-not1* hybridization signals present in the parenchyma were reduced but did not completely disappear by day 3 post-irradiation, it is likely that *Smed-not1* is expressed in neoblasts and their recent progeny, but also other post-mitotic cells. These data indicates that *Smed-not1* is broadly expressed throughout the planarian body, consistent with a housekeeping function, in a pattern that includes neoblasts, their progeny as well as differentiated cells. Investigation of Not1 expression in previous transcriptome based studies of mRNAs expressed in neoblasts is consistent with this expression pattern (Figure S2).

### *Smed-not1* is required for planarian regeneration and homeostatic cell turnover

**Figure 2:**
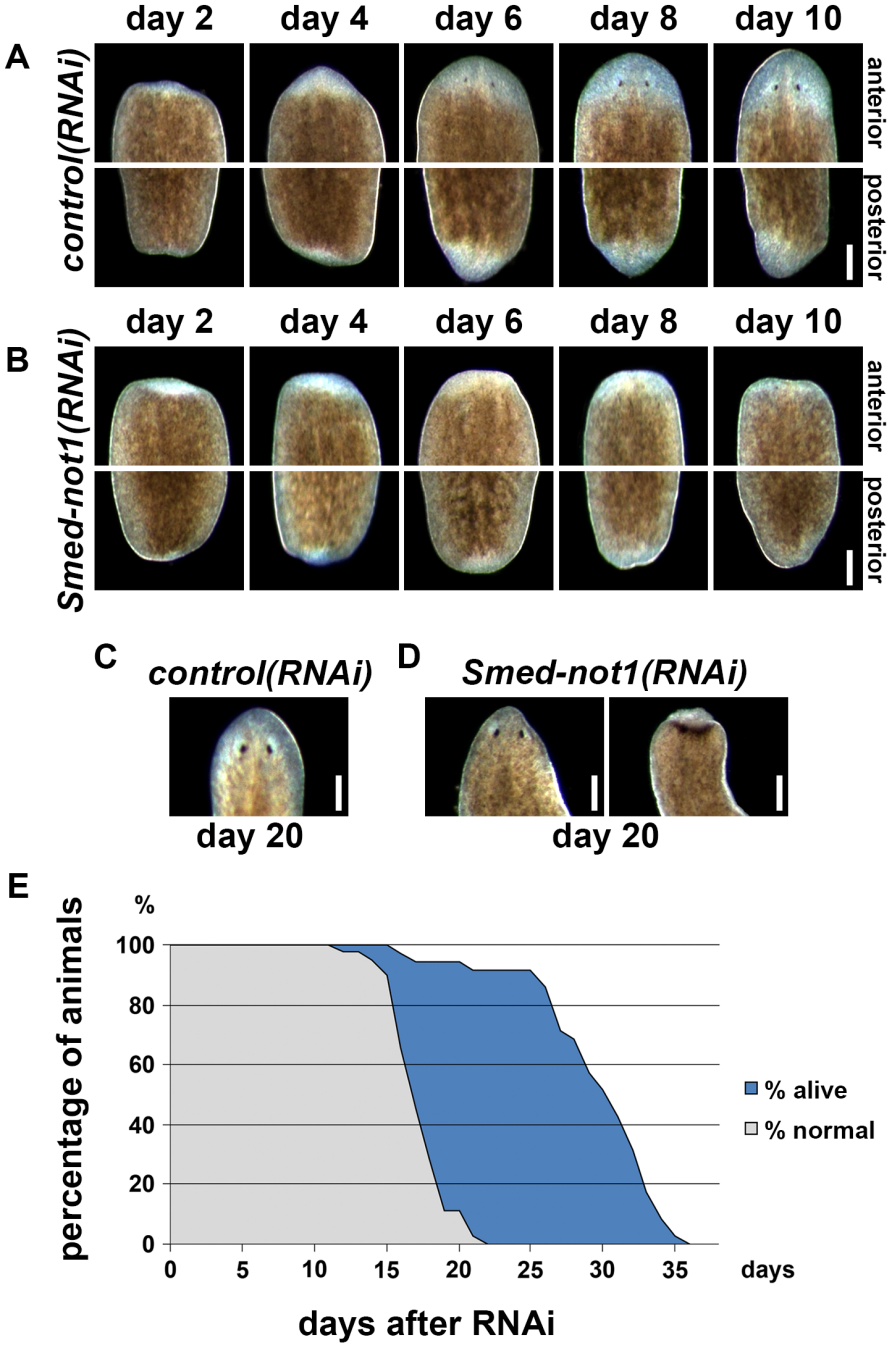
*Smed-not1* is required for planarian regeneration and homeostatic cell turnover. (A–B) *Control(RNAi)* (A) and *Smed-not1(RNAi)* (B) animals cut 5 days after RNAi, and monitored every 2 days after transection. Top panels: anterior wounds; bottom panels: posterior wounds. *Smed-not1* animals are able to produce blastemas (white tissue) in both anterior and posterior wounds but fail to regenerate. (C–D) Intact *control(RNAi)* (C) and *Smed-not1(RNAi)* (D) animals 20 days after RNAi, anterior side. *Smed-not1* animals 20 days after RNAi display variable levels of head regression defects. (E) Survival curve of *Smed-not1* animals (N = 40), indicating the percentage of animals without any defects (grey) and the percentage alive (blue). All *control(RNAi)* animals survived without defects for more than 40 days (N = 40). Scale bars: 500 µm. See also Figure S3.

We then analyzed the function of *Smed-not1* by RNAi experiments. All *Smed-not1(RNAi)* animals displayed abrogated regeneration capacities and eventually died. They were able to produce both anterior and posterior regeneration blastemas, but never completed the regenerative process (Figure 2B, vs. Figure 2A). In order to test if the formation of a regeneration blastema depended on the time of transection, we cut animals at 1, 3, 5, 10 and 15 days after *Smed-not1(RNAi)* treatment (Figure S3). We found that animals were able to produce a regeneration blastema at all-time points, however animals cut earlier produced larger blastemas. All blastemas of *Smed-not1(RNAi)* worms eventually regressed. The ability of *Smed-not1(RNAi)* animals to produce a large regeneration blastema at early time points after RNAi suggests that mitotic neoblasts, the source of blastema cells, are still present and proliferating.

We then analyzed the phenotype of intact *Smed-not1(RNAi)* animals. We found that *Smed-not1(RNAi)* worms presented head regression (Figure 2D, vs. Figure 2C) [43], [44], 64,65, a symptom of interrupted homeostatic cell turnover. A survival curve during which the onset of tissue homeostasis defects was recorded (N = 40) demonstrated temporal phenotypic variability (Figure 2E). In *Smed-not1(RNAi)* animals these defects were seen first at 15 days after RNAi, and in the majority of animals after 20 days (Figure 2C). Variable degrees of head regression were also observed after 20 days of RNAi (Figure 2C, also see Figure S3). By day 22 all animals had defects, showing complete penetrance of *Smed-not1* RNAi. All animals died by day 36 after dsRNA delivery, with the majority of deaths occurring between day 26 and day 34 (Figure 2E). All *control(RNAi)* animals survived without any defect for >35 days, however. Together, these results demonstrate that *Smed-not1* is needed for regeneration and homeostatic cell turnover in *S. mediterranea*.

### *Smed-not1(RNAi)* animals maintain proliferative neoblasts

**Figure 3:**
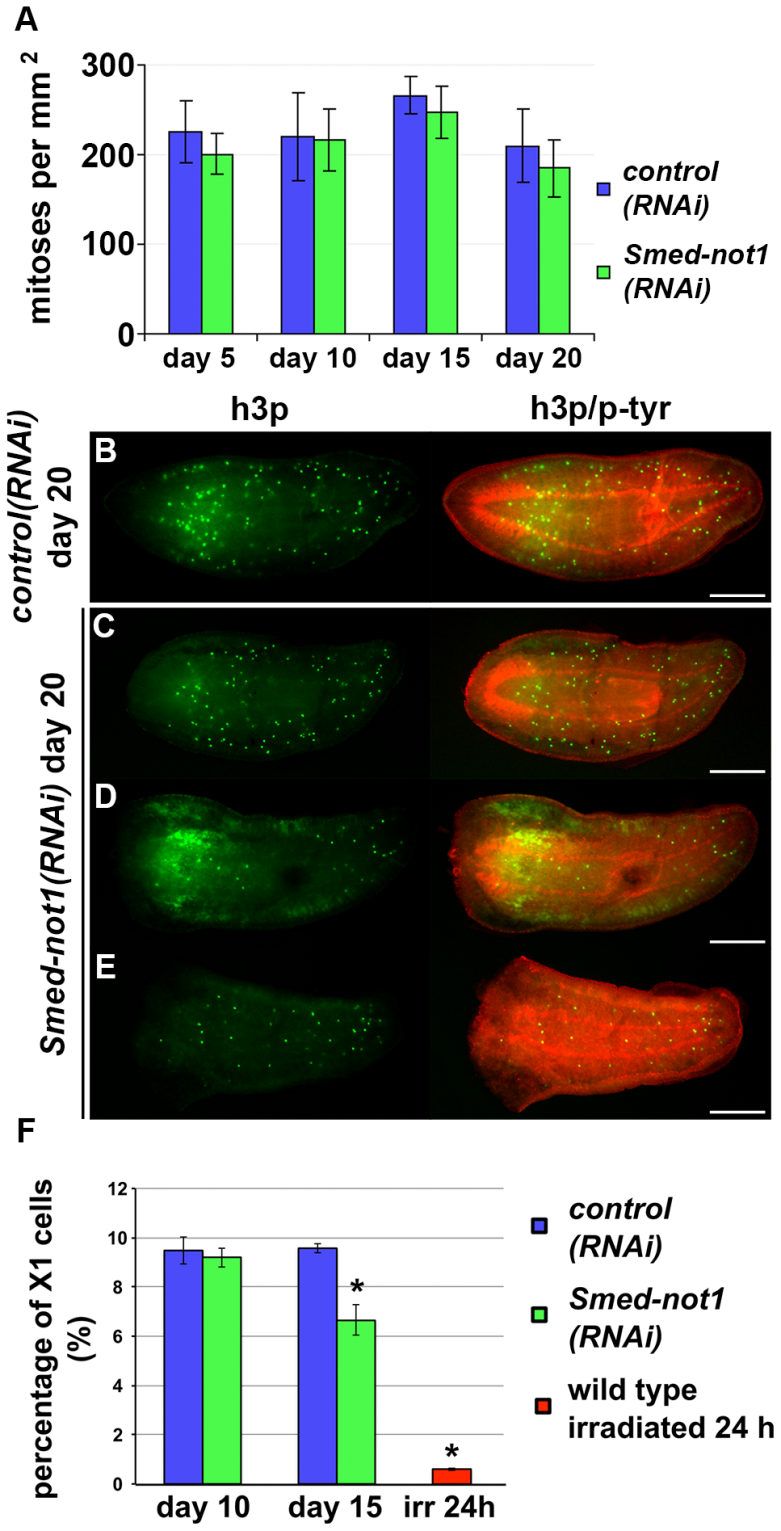
*Smed-not1(RNAi)* animals maintain mitotic neoblasts. (A) Quantification of mitosis by counting of h3p-positive cells in whole mount immunohistochemistry on *control(RNAi)* and *Smed-not1(RNAi)* animals 5, 10, 15 and 20 days after RNAi (N = 7 per time point). No significant differences are detected. Representative *control(RNAi)* (B) and different *Smed-not1(RNAi)* worms (C–E) 20 days after RNAi, immunostained with the mitotic marker h3p (h3p, green) and counterstained with phospho-tyrosine (p-tyr, red) in order to show head regression defects. *Smed-not1(RNAi)* animals still display detectable mitotic cells, even as head regression defects occur. The number of mitotic cells detected is smaller in the animals with the most severe head regression defects (D–E). Anterior is to the left. Scale bars: 500 µm. (F) Quantification of FACS sorted X1 cells in *control(RNAi)* and *Smed-not1(RNAi)* animals 10 and 15 days after RNAi, and wild type irradiated animals. While no significant differences are observed 10 days after knock down, *Smed-not1(RNAi)* animals show a reduced but significant decrease in percentage of X1 cells. Error bars represent standard deviation and asterisks represent statistical significance. See also Figure S4.

Next, we analyzed the mitotic marker phospho-histone-H3 (h3p) in *Smed-not1(RNAi)* animals at 5, 10, 15 and 20 days after RNAi. Up to 15 days all (N = 7 per time point) had mitotic neoblasts comparable in numbers to those of *control(RNAi)* animals (Figure 3A). After 20 days all animals still had mitotic cells, but at variable levels, in agreement with our earlier phenotypic characterisation (Figure 3B–E). Most had normal levels (Figure 3C, vs. Figure 3B), although animals with more severe head regression defects showed a visible reduction in the mitotic levels (Figure 3D–E, vs. Figure 3B–C), but overall the reduction in mitoses was not statistically significant. These experiments show that *Smed-not1(RNAi)* worms have mitotic cells, even as head regression defects progress. Significantly, similar defects are seen in irradiated worms only weeks after complete loss of mitotic activity. Therefore, we interpret our data as showing that effects on stem cell proliferation are not the primary cause of the *Smed-not1(RNAi)* phenotype, instead implicating neoblast differentiation impairment as responsible for regenerative failure, head regression and other defects.

By performing FACS experiments we found that *Smed-not(RNAi)* led to a moderate reduction of the sorted irradiation sensitive X1 cells, which primarily contains neoblasts, at 15 days but not at 10 days (Figure 3F. Figure S4A–B). Even though no significant decrease of h3p cells was detected at this time point we interpret our FACS data as more sensitive and conclude that both methods consistently detect large numbers of proliferating neoblasts 15 days after *Smed-not1* dsRNA administration, further implicating neoblast differentiation defects instead.

### Dynamics of neoblast cells and their progeny in *Smed-not1(RNAi)* animals

**Figure 4:**
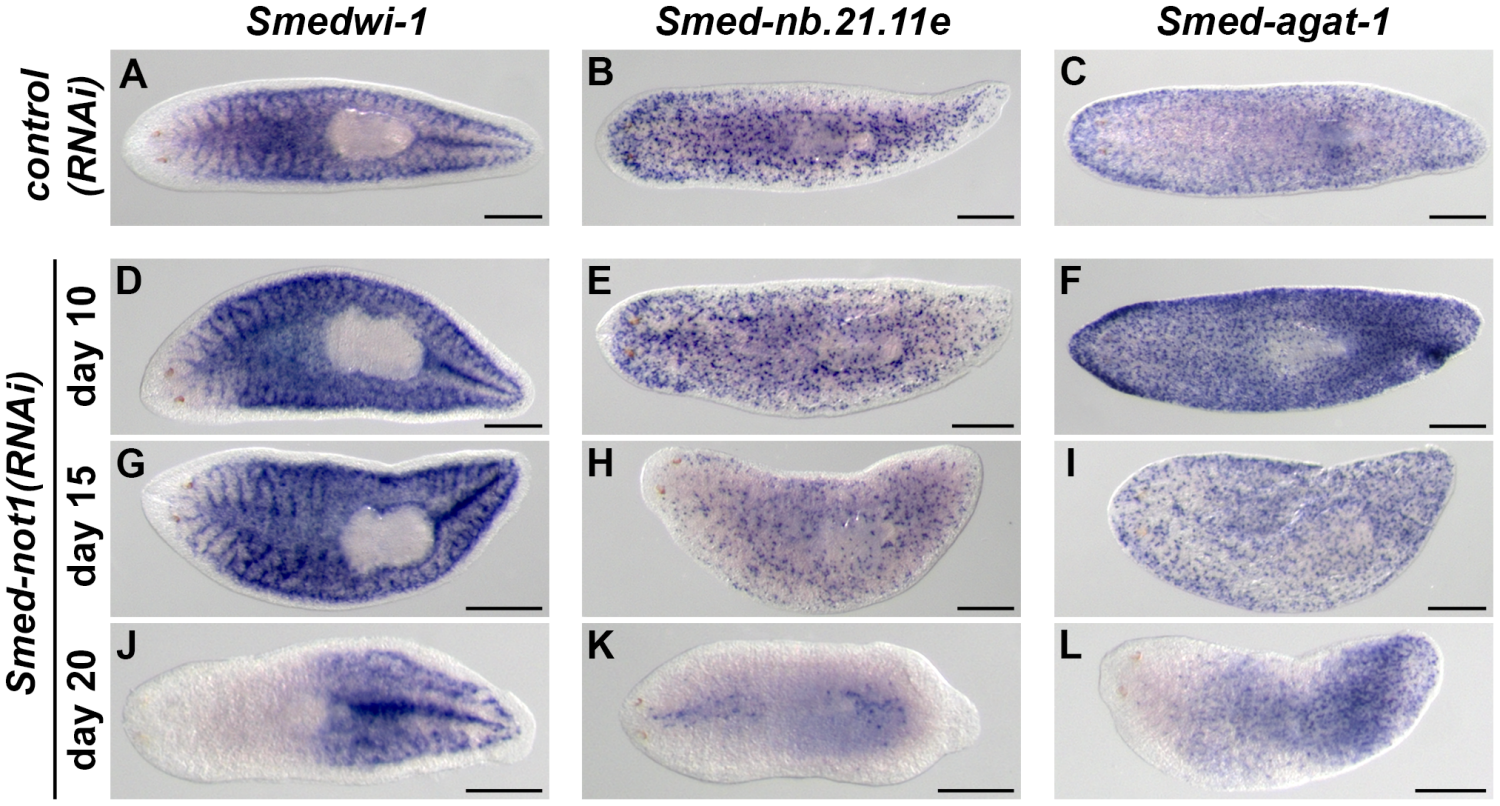
Dynamics of neoblasts and their progeny in *Smed-not1(RNAi)* animals. (A–L) WMISH of the neoblast marker *Smedwi-1* (A, D, G, J), the early neoblast progeny marker *Smed-nb.21.11e* (B, E, H, K) and the late neoblast progeny marker *Smed-agat-1* (C, F, I, L) in *control(RNAi)* animals (A–C) and *Smed-not1(RNAi)* animals 10 (D–F), 15 (G–I) and 20 (J–L) days after RNAi. *Smed-not1(RNAi)* animals have detectable expression of *Smedwi-1* in all time points (A, D, G, J), although a decline in the level of *Smedwi-1* signals is detected 20 days after RNAi (J). The dynamics of progeny markers is also abnormal, with a progressive decline of *Smed-nb.21.11e* signals (H, K) and an accumulation (F) followed by a decline (I, L) of *Smed-agat-1* signals. Anterior is to the left. Scale bars: 500 µm. See also Figure S5.

In order to monitor the behaviour of neoblasts and their post-mitotic progeny during progression of the *Smed-not1* knock down phenotype we analysed the expression of neoblast and progeny markers [66]. *Smedwi-1*, a marker of neoblasts, *Smed-nb.21.11e*, a marker of early neoblast progeny, and *Smed-agat-1*, a marker of late neoblast progeny, were analyzed in *control(RNAi)* worms (Figure 4A–C) and *Smed-not1(RNAi)* worms after 10 (Figure 4D–F), 15 (Figure 4G–I), and 20 (Figure 4J–L, Figure S5A–F) days of RNAi. Only one time point (10 days) is shown for *control(RNAi)* worms, since no differences were observed between time points. *Smedwi-1* expression was qualitatively the same after 10 and 15 days of *Smed-not1(RNAi)*, (Figure 4D, G, vs. Figure. 4A), but clearly reduced to a variable extent after 20 days (Figure 4J vs. Figure 4A, D and G; Figure S5A vs. Figure S5D); some animals had nearly normal expression while *Smedwi-1* expression was severely reduced in those with the most severe head regression defects. These results, like those above, suggest that prominent stem cell loss occurs only when *Smed-not1(RNAi)* animals begin to regress the head and to die, again implicating differentiation impairment instead of proliferation or self-renewal as a primary cause for regenerative failure.

Expression of *Smed-nb.21.11e*, a marker of early neoblast progeny, looked broadly equivalent to that of control worms in animals fixed 10 days after *Smed-not1* dsRNA delivery (Figure 4E, vs. Figure 4B) but a clearly visible progressive decrease in the number of *Smed-nb.21.11e*-positive cells was detected after 15 (Figure 4H, vs. Figure 4B) or 20 (Figure 4K, vs. Figure 4B) days after RNAi. After 20 days of *Smed-not1* knock down animals had only a few remaining *Smed-nb.21.11e*-positive cells (Figure S5E). These results show that clearly visible decreases in early progeny cell number precede the prominent decrease in neoblasts themselves. When we checked the expression of *Smed-agat-1*, a marker of late neoblast progeny, we observed a consistent qualitative increase in *Smed-agat-1*-positive cells in *Smed-not1(RNAi)* worms after 10 days (Figure 4F vs. Figure 4C). At later time points, however, the number of *Smed-agat-1*-positive cells also progressively declined (Figure 4I, L, vs. Figure 4C). Again, we observed a considerable variability in *Smed-not1(RNAi)* worms after 20 days (Figure S5F). However, in all animals with a clear decrease in the number of *Smed-agat-1*-positive cells this was particularly apparent in the anterior region, a characteristic feature of *Smed-agat-1*-positive cell depletion upon neoblast elimination by irradiation or perturbation by RNAi [50], [64], [65], [67].

Therefore, neoblasts are abundant 15 days after RNAi, and only clearly start to be depleted later, coinciding with the onset of head regression defects. Similar defects are observed after irradiation, however, these take >10 days to manifest after elimination of mitotic activity. In contrast *Smed-not1(RNAi)* animals display these defects when neoblasts are still present. These results suggest that a primary defect in neoblast differentiation, rather than neoblast maintenance, causes failure in tissue homeostasis. In support of this idea, altered stem cell progeny numbers precede the disappearance of *Smedwi-1* signals and mitotic activity. This alteration can be observed as early as 10 days after RNAi in the case of *Smed-agat-1*-positive cells and 15 days for *Smed-nb.21.11e*-positive cells, which are clearly depleted at this time point.

To further test this, we compared the dynamics of the neoblast and progeny cell markers in *Smed-not1(RNAi)* animals to those of *Smedwi-2(RNAi)* animals, in which neoblast differentiation is abrogated [44]. *Smedwi-1*, *Smed-nb.21.11e* and *Smed-agat-1* were expressed in *Smedwi-2(RNAi)* animals with very similar dynamics to *Smed-not1(RNAi)* animals (Figure S5G–R). Taken together, these results show that, similar to *Smedwi-2* RNAi, *Smed-not1* RNAi impairs neoblast differentiation with proliferation only affected in a later time point.

### *Smed-not1(RNAi)* animals have abnormal numbers of *Smed-agat-1* transcripts and *Smed-agat-1*-positive cells

**Figure 5:**
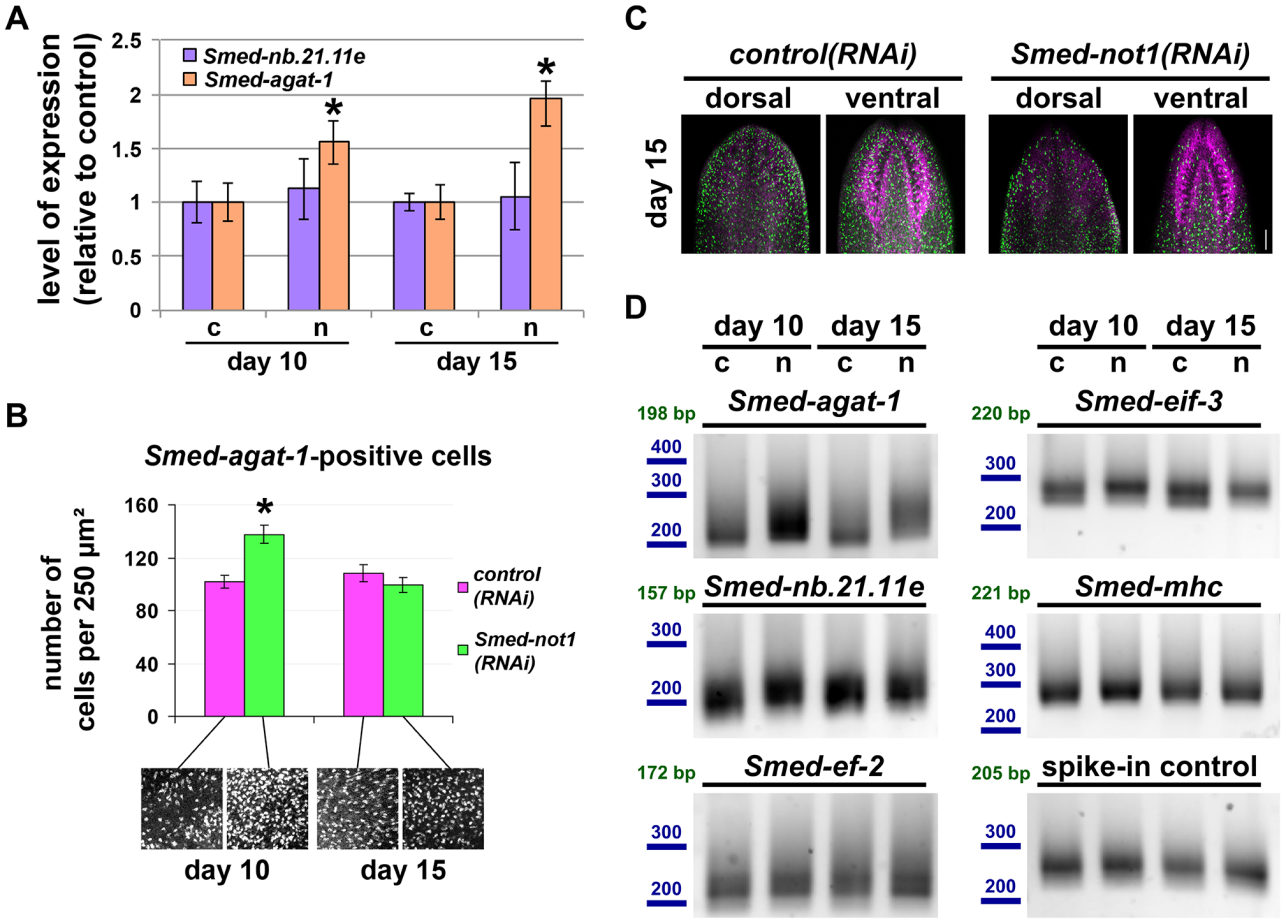
*Smed-not1(RNAi)* animals have increasing numbers of *Smed-agat-1* transcripts with increased frequency of long poly(A) tails but decreasing numbers of *Smed-agat-1*-positive cells. (A) Quantification of the level of expression by qRT-PCR of the progeny markers *Smed-nb.21.11e* and *Smed-agat-1* in *Smed-not1(RNAi)* animals 10 and 15 days after RNAi, normalized expression and relative to respective *control(RNAi)* samples. Error bars represent standard deviation, asterisks represent statistical significance. *Smed-agat-1* transcripts accumulate progressively after 10 and 15 days of RNAi. (B) Quantification of the number of *Smed-agat-1*-positive cells in *control(RNAi)* and *Smed-not1(RNAi)* worms 10 and 15 days after RNAi. Animals (N = 8 per time point and treatment) were stained by FWMISH of *Smed-agat-1* and analyzed by confocal microscopy. Multiple squares corresponding to 250 µm^2^ in both the dorsal and ventral parts of the animals were selected for counting along the length of the animals. 130 squares were counted, 4 representative squares are shown. Error bars represent standard error of the mean, asterisk represents statistical significance. A significant accumulation of *Smed-agat-1*-positive cells is detected in *Smed-not1(RNAi)* animals 10 days after RNAi but a decrease is observed at 15 days (C) Confocal Z-projection of FWMISHs of *Smed-agat1* in *control(RNAi)* (top panels) and *Smed-not1(RNAi)* (bottom panels) animals corresponding to anterior dorsal (left) and anterior ventral (right) regions of representative worms. *Smed-agat-1*-positive cells are shown in green, nuclei counterstaining in magenta. Scale bar: 100 µm. (D) PAT assays reflecting the distribution of mRNA poly(A) tail lengths for *Smed-agat-1* and *Smed-nb.21.11e*, the housekeeping mRNAs *Smed-ef-2* and *Smed-eif-3*, the tissue specific mRNA *Smed-mhc* and a spiked-in control mRNAs in *control(RNAi)* (c) and *Smed-not1(RNAi)* (n) animals 10 and 15 days after RNAi. Size markers used are represented in blue, the theoretical length of the amplicon corresponding to the deadenylated mRNA species given the primers used in each assay is given in green. Products above this length originate from polyadenylated mRNA molecules. Marked differences in poly(A) tail length distribution are detected for *Smed-agat-1*, showing an increased frequency of long poly(A) tails after *Smed-not1* knock down. Slight differences are observed for *Smed-nb.21.11e* and *Smed-eif-3*.

In order to achieve a quantitative measure of stem cell progeny mRNA levels in *Smed-not1(RNAi)* animals we performed quantitative real time PCR experiments (qRT-PCR) of *Smed-nb.21.11e* and *Smed-agat-1* transcripts in *Smed-not1(RNAi)* worms. We focused on earlier time points of 10 and 15 days after RNAi. qRT-PCR showed that *Smed-nb.21.11e* levels in whole animals were similar to those of *control(RNAi)* after 10 and 15 days of dsRNA administration (Figure 5A). However, *Smed-agat-1* mRNA levels increased by day 10 and were almost two-fold higher after 15 days. Since this result did not correlate with what we observed by colorimetric WMISH, we quantified *Smed-agat-1*-positive cells by fluorescent WMISH (FWMISH). A significant increase in the numbers of *Smed-agat-1*-positive cells was found in *Smed-not1(RNAi)* animals 10 days after dsRNA administration (Figure 5B), but their numbers declined to control levels after 15 days. These data confirmed the qualitative data from colorimetric WMISH (Figure 4). The distribution of *Smed-agat-1*-positive cells in *Smed-not1(RNAi)* animals 15 days after dsRNA delivery was different from controls, with less *Smed-agat-1*-positive cells in the anterior part of the worm (Figure 5C). This revealed a stark discordance between the number of *Smed-agat-1*-positive cells and the level of *Smed-agat-1* mRNA. We conclude that *Smed-agat-1* transcripts are accumulating in decreasing numbers of *Smed-agat-1*-positive cells after 15 days of *Smed-not1* knock down, and that each *Smed-agat-1*-positive cell contains an increased average number of *Smed-agat-1* transcripts. A similar, but less pronounced, process could also explain reduced numbers of *Smed-nb.21.11e* positive cells and discordant stable levels of this transcript, which do not drop by day 15.

Given that the CCR4-NOT complex is known to regulate gene expression through its deadenylating activity we wished to ascertain if it could be directly responsible of the discordance between *Smed-agat-1* positive cell number and mRNA levels. If an increase in mRNA levels is caused by impaired deadenylation and subsequent degradation we would expect to observe increased frequency of long poly(A) tail lengths. Using a poly(A) tail length (PAT) assay [68]–[71], in whole worms we observed that this was the case for both *Smed-agat-1* and *Smed-nb.21.11.e* (Figure 5D), with the first giving starker differences, while control mRNAs *Smed-eif-3*, *Smed-mhc*, or *Smed-ef-2* were only mildly affected or not affected at all. In addition a spike-in control of exogenous mRNA showed equal poly(A) tail length distribution across samples (Figure 5D), showing that the differences observed are present in our different planarian mRNA samples and not introduced by the PAT assay technique.

### *Smed-not1(RNAi)* animals have increased levels of transcripts expressed in stem cells with increased frequency of long poly(A) tails

**Figure 6:**
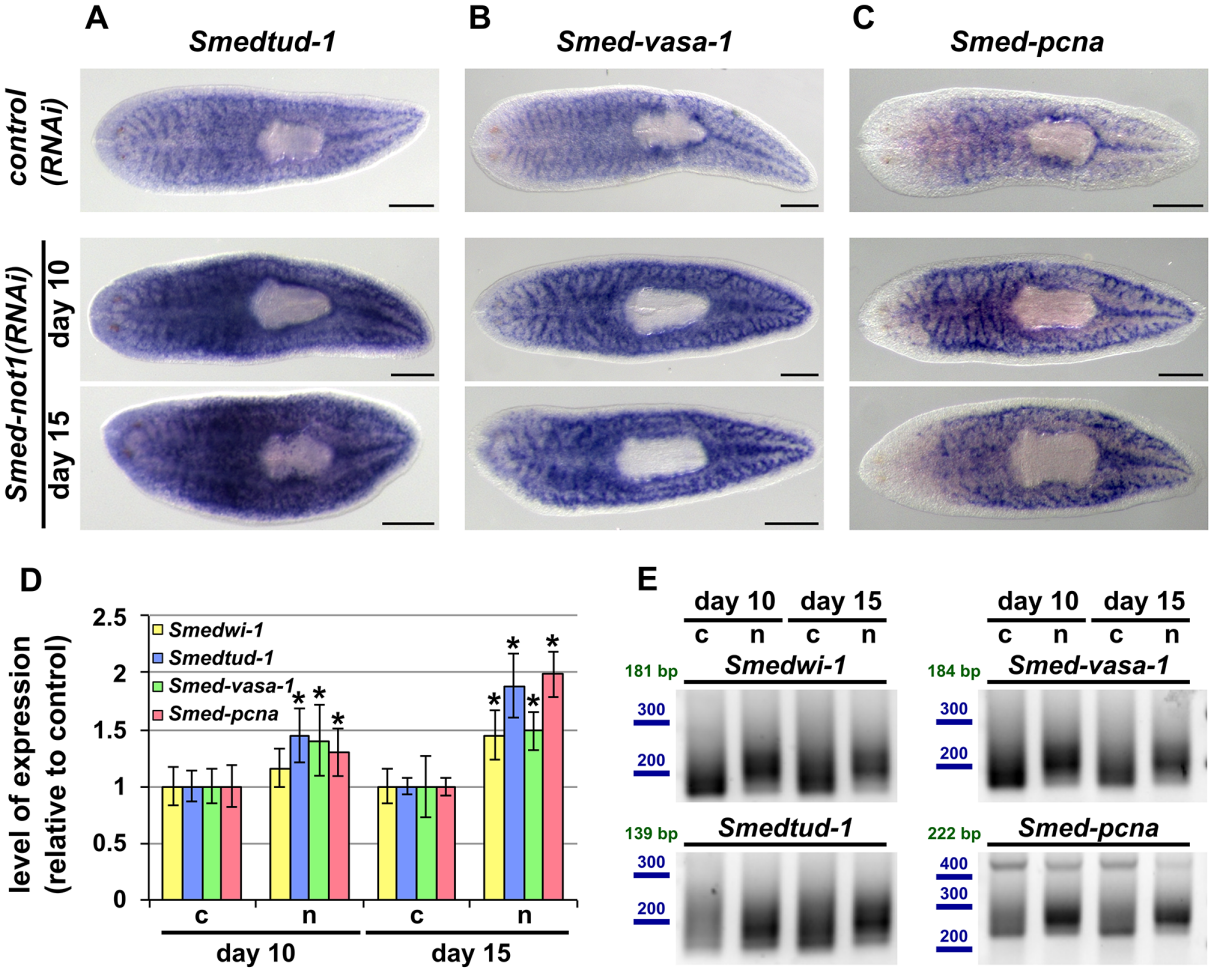
*Smed-not1(RNAi)* animals have increased levels of neoblast transcripts with increased frequency of long poly(A) tails. (A–I) WMISH of the neoblast marker *Smedtud-1* (A), *Smed-vasa-1* (B) and *Smed-pcna* (C) in *control(RNAi)* animals (upper panels) and *Smed-not1(RNAi)* animals 10 (middle panels) and 15 (bottom panels) days after RNAi, showing normal expression of these mRNAs after *Smed-not1* knock down, though qualitative differences in the level of expression are suggested. Anterior is to the left. Scale bars: 500 µm. (D) Quantification of the level of expression by qRT-PCR of the neoblast markers *Smedwi-1*, *Smedtud-1*, *Smed-vasa-1* and *Smed-pcna* in *control(RNAi)* (c) and *Smed-not1(RNAi)* animals (n) 10 and 15 days after RNAi, normalized expression and relative to respective *control(RNAi)* samples. Error bars represent standard deviation, asterisks represent statistical significance. *Smedtud-1*, *Smed-vasa-1* and *Smed-pcna* transcripts accumulate progressively after 10 and 15 days of RNAi, while *Smedwi-1* only accumulates significantly after 15 days. (E) PAT assays reflecting the distribution of mRNA poly(A) tail lengths for *Smedwi-1*, *Smedtud-1*, *Smed-vasa-1* and *Smed-pcna* in *control(RNAi)* (c) and *Smed-not1(RNAi)* animals (n) 10 and 15 days after RNAi. Size markers used are represented in blue, the theoretical length of the amplicon corresponding to the deadenylated mRNA species given the primers used in each assay is given in green. Marked differences in poly(A) tail length distribution are detected for all four mRNAs, showing an increased frequency of long poly(A) tails after *Smed-not1* knock down.

Next, we found that WMISH analysis of neoblast markers, *Smedtud-1*, *Smed-vasa-1* and *Smed-pcna*, was suggestive of increased levels of these transcripts in *Smed-not1(RNAi)* worms, with qualitatively visible differences after both 10 and 15 days after RNAi treatment (Figure 6A–C). These data further demonstrate that neoblast maintenance is not affected by *Smed-not1* knock down at these time points.

Since WMISH does not provide a quantitative measure of mRNA levels we quantified these differences by qRT-PCR experiments on RNA from *Smed-not1(RNAi)* animals. *Smedtud-1*, *Smed-vasa-1* and *Smed-pcna* all progressively increased to levels approximately 50% and 100% higher than those of *control(RNAi)* after 10 and 15 days, respectively (Figure 6D). *Smedwi-1* transcript levels were also significantly increased in whole animals after 15 days (Figure 6D). Collectively, these results demonstrate that *Smed-not1* RNAi knock down leads to an increase in mRNA levels in several genes expressed in neoblasts and their progeny.

Given the known conserved function of the CCR4-NOT complex in regulating mRNA levels through targeted deadenylation we performed PAT assays on the set of neoblast markers and on the samples above (Figure 5D). In all cases *Smed-not1* knock down resulted in increased average poly(A) tail length (Figure 6E), demonstrating that increased transcript levels correlate with a failure in deadenylation. These data confirm that knock down of the CCR4-NOT complex subunit *Smed-not1* leads to increased transcript levels of genes known to be key to neoblast function, by blocking their deadenylation and subsequent degradation.

### Upregulation and increased polyadenylation of *Smedtud-1*, *Smed-vasa-1* and *Smed-pcna* occurs specifically in stem cells

Since many neoblast mRNAs are also expressed in differentiated cells (e. g. *Smedtud-1* and *Smed-vasa-1* are also prominently expressed in the CNS, Figure 1B–C), the increased mRNA levels we detected could arise from a response in differentiated cells alone, stem cells alone or from both differentiated and stem cells. To distinguish these possibilities, we planned to use an irradiation approach to remove all neoblasts and then measure transcript levels in *Smed-not1(RNAi)* and *control(RNAi)* worms by qRT-PCR. We reasoned that if transcript accumulation was indeed limited to stem cells then mRNA levels of these transcripts after irradiation would be equal in both irradiated *control(RNAi)* and irradiated *Smed-not1(RNAi)* samples.

*Smedtud-1* and *Smed-vasa-1* are expressed in the CNS to levels that amount respectively to roughly 70% and 40% of their total expression, according to our previous neoblast profiling by a combinatorial RNA-seq, RNA interference and irradiation approach [50]. We confirmed this by qRT-PCRs (Figure S5A) and observed that *Smed-pcna* expression amounts to roughly only 10% of its normal expression 24 hours after irradiation (Figure S6A), indicating that 24 hours of irradiation suffice to eliminate around 90% of neoblasts. Conversely, most neoblast progeny survive beyond 1 day post-irradiation, as measured by our qRT-PCR experiments with the markers *Smed-nb.21.11e* and *Smed-agat1* (Figure S6B) and consistent with previously published data [50], [66], making then 24 hours after irradiation the ideal time point to perform our experiment.

**Figure 7:**
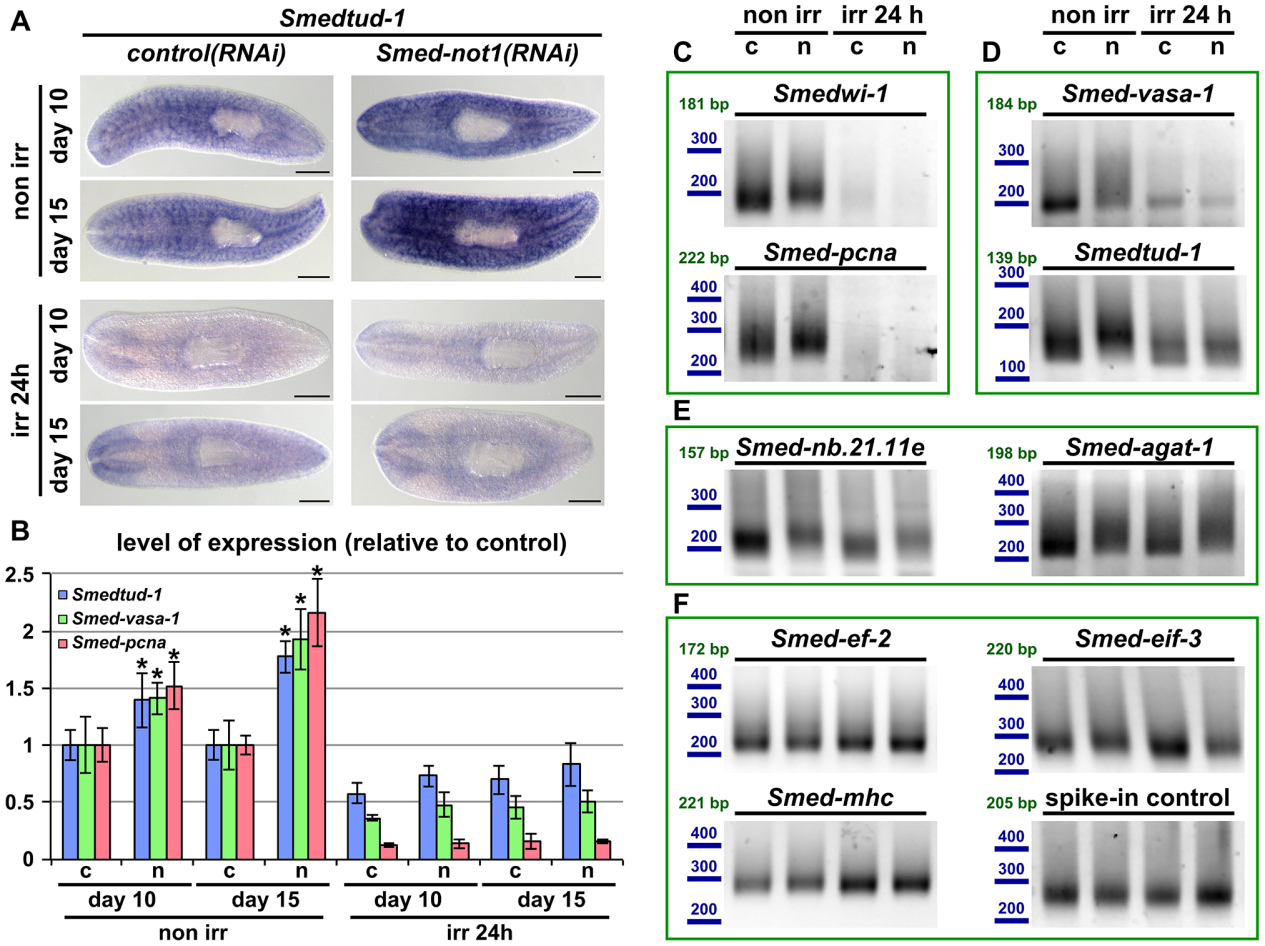
Increased levels of neoblast transcripts and their increased frequency of long poly(A) tails are irradiation sensitive. (A) WMISH of the neoblast marker *Smedtud-1* in *control(RNAi)* animals (left panels) and *Smed-not1(RNAi)* animals (right panels) non irradiated (top panels) and irradiated 24 hours (bottom panels) prior to data collection time points 10 and 15 days after RNAi. A consistent qualitative difference is observed in non irradiated animals, however, no qualitative differences are observed in irradiated animals, showing that *Smed-not1(RNAi)* animals do not overexpress ectopically *Smedtud-1*. Anterior is to the left. Scale bars: 500 µm. (B) Quantification of the level of expression by qRT-PCR of the neoblast markers *Smedtud-1*, *Smed-vasa-1* and *Smed-pcna* in *control(RNAi)* (c) and *Smed-not1(RNAi)* (n) animals non-irradiated and irradiated 24 hours prior to data collection time points 10 and 15 days after RNAi, normalized expression and relative to respective *control(RNAi)* samples. Error bars represent standard deviation, asterisks represent statistical significance. *Smedtud-1*, *Smed-vasa-1* and *Smed-pcna* transcripts accumulate progressively after 10 and 15 days of RNAi, but this accumulation is eliminated 24 hours post irradiation, with *Smed-not1(RNAi)* irradiated animals showing levels similar to *control(RNAi)* irradiated animals. (C–F) PAT assays reflecting the distribution of mRNA poly(A) tail lengths for the neoblast specific mRNAs *Smedwi-1* and *Smed-pcna* (C), the neoblast and CNS expressed mRNAs *Smed-vasa-1* and *Smedtud-1* (D), the progeny specific mRNA *Smed-nb.21.11e* and *Smed-agat-1* (E) and the housekeeping and tissue specific mRNAs *Smed-ef-2*, *Smed-eif-3*, *Smed-mhc* and a spiked-in control in *control(RNAi)* (c) and *Smed-not1(RNAi)* (n) animals non irradiated and irradiated 24 hours prior to data collection time points 15 days after RNAi. Size markers used are represented in blue, the theoretical length of the amplicon corresponding to the deadenylated mRNA species given the primers used in each assay is given in green. Neoblast specific markers are not detected after irradiation (C). The marked differences in poly(A) tail length distribution detected for the neoblast and CNS mRNAs *Smed-vasa-1* and *Smedtud-1* are eliminated by irradiation, showing that the fractions of these mRNA populations localised in the CNS show no differences after *Smed-not1* knock down (D). The differences in poly(A) tail length distribution detected for the progeny specific mRNAs *Smed-nb.21.11e* and *Smed-agat-1* are not affected by irradiation, as these cells are not eliminated after 24 hours of irradiation (E). No differences are detected for *Smed-ef-2*, *Smed-eif-3*, *Smed-mhc* and a spike-in control RNA.

Therefore, in order to find out if the increased levels of neoblast mRNAs originate in neoblasts or instead in the CNS or elsewhere, we then used this irradiation approach in *Smednot-1(RNAi)* animals and compared them to *control(RNAi)* animals. We irradiated both *Smednot-1(RNAi)* and *control(RNAi)* animals at 9 and 14 days after dsRNA administration, 24 hours before the data collection time points of 10 and 15 days respectively. We then examined the expression pattern of *Smedtud-1* by WMISH. This confirmed our qRT-PCR experiments, and was consistent with our previous WMISH results (Figure 7A). Non irradiated *Smed-not1(RNAi)* animals showed qualitatively more intense expression of *Smedtud-1*, However, all animals that were irradiated 24 hours prior to fixation showed an identical expression pattern of *Smedtud-1*, with similar levels of signal detected only in the CNS to their *control(RNAi)* irradiated counterparts (Figure 7A). This experiment shows that *Smedtud-1* is not ectopically expressed in other tissues or organs after *Smed-not1* knock down, since this ectopic expression should be visible either in non irradiated or irradiated samples, and suggests instead that the increased levels of *Smedtud-1* come from an accumulation of this transcript in neoblasts.

We performed qRT-PCR and confirmed that *Smedtud-1*, *Smed-vasa-1* and *Smed-pcna* mRNA levels increased progressively in *Smed-not1(RNAi)* animals (Figure 7B, left) but both *control(RNAi)* and *Smed-not1(RNAi)* animals irradiated 24 hours previously contained similar levels of all three transcripts (Figure 7B, right). Similar to results for wild type worms (Figure S6A), the levels of the *Smedtud-1*, *Smed-vasa-1* and *Smed-pcna* transcripts were respectively around 70%, 40% and 10% of the expression in non-irradiated *control(RNAi)* animals. Therefore we conclude that the overexpression observed in *Smed-not1(RNAi)* animals for these three transcripts disappears 24 hours after irradiation, and is therefore located in irradiation-sensitive neoblasts, rather than elsewhere in the body.

To implicate the CCR4-NOT complex-mediated targeted mRNA degradation directly in this effect we performed PAT assays on non irradiated and irradiated samples and observed that the distribution of poly(A) tail lengths of all these neoblast mRNAs was increased in *Smed-not1(RNAi)* animals, but this effect was removed by irradiation (Figure 7C and D). In the case of neoblast specific *Smedwi-1* and *Smed-pcna* no signal was detected after irradiation (Figure 7C). In the case of *Smed-vasa-1* and *Smedtud-1* signal was detectable after irradiation, reflecting expression in the CNS, but these transcripts did not show any increase in poly(A) tail length distribution after *Smed-not1* knock down (Figure 7D). These data confirm that effects on mRNA levels for these important neoblast genes are confined to stem cells. As an additional control we also measured *Smed-nb.21.11e* and *Smed-agat-1* poly(A) lengths (Figure 7E). 24 hours after irradiation cells expressing these transcripts are still present and the increase in poly(A) tail length caused by *Smed-not1* knock down is still evident. This confirms that irradiation itself does not cause the absence of detected differences in poly(A) tail length *per se*, but by removing the cycling neoblasts. The poly(A) tail length distribution of control mRNAs were not affected by irradiation (Figure 7F).

**Figure 8:**
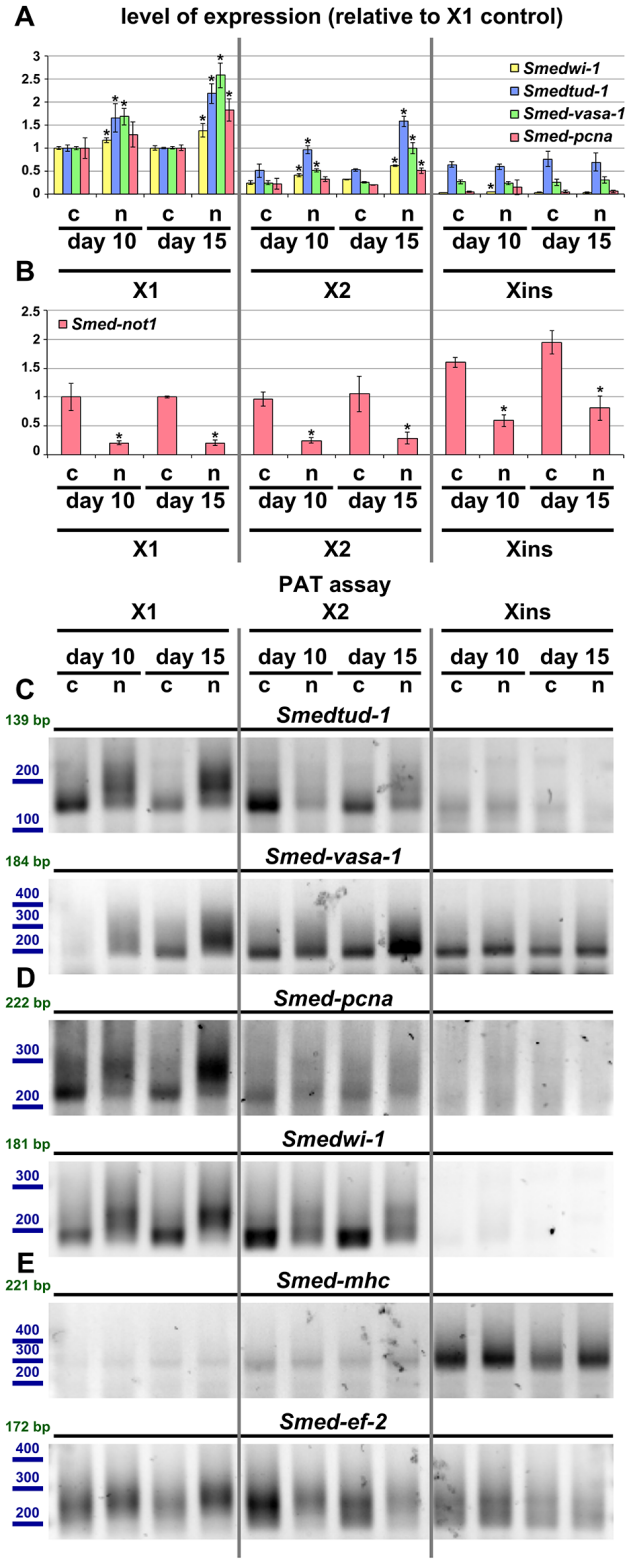
Increased level of transcripts and increased frequency of long poly(A) tails are restricted to neoblast-containing cell populations. (A–B) Quantification of the level of expression by qRT-PCR of the neoblast markers *Smedwi-1*, *Smedtud-1*, *Smed-vasa-1* and *Smed-pcna* (A) and *Smed-not1* (B) in FACS sorted populations X1, X2 and Xins in *control(RNAi)* (c) and *Smed-not1(RNAi)* (n) animals 10 and 15 days after RNAi, normalized expression and relative to respective X1 *control(RNAi)* samples. (A) *Smedwi-1*, *Smedtud-1*, *Smed-vasa-1* and *Smed-pcna* transcripts accumulate progressively after 10 and 15 days of RNAi in X1 and X2 cells, the two fractions that contain neoblasts to different extents, but this accumulation is not observed in Xins cells, which contain differentiated cells exclusively, including CNS cells. (B) *Smed-not1* is significantly depleted across all three cell fractions, showing that the absence of accumulation and increased frequency of long poly(A) tails of neoblast mRNAs that are expressed also in CNS is not due to absence of effective gene knock down in differentiated cells. Error bars represent standard deviation and asterisks represent statistical significance in A–B (C–E) PAT assays reflecting the distribution of mRNA poly(A) tail lengths for the neoblast and CNS expressed mRNAs *Smedtud-1* and *Smed-vasa-1* (C), the neoblast specific mRNAs *Smed-pcna* and *Smedwi-1* (D) and the housekeeping and tissue specific mRNAs *Smed-mhc* and *Smed-ef-2* (E) in FACS sorted populations X1, X2 and Xins from *control(RNAi)* (c) and *Smed-not1(RNAi)* (n) animals 10 and 15 days after RNAi. Size markers used are represented in blue, the theoretical length of the amplicon corresponding to the deadenylated mRNA species given the primers used in each assay is given in green. (C). The marked differences in poly(A) tail length distribution detected for the neoblast and CNS mRNAs *Smed-vasa-1* and *Smedtud-1* are only detected in X1 and X2 but not in Xins FACS sorted populations, showing that the fractions of these mRNA populations localised in the CNS show no differences after *Smed-not1* knock down. (D) The marked differences in poly(A) tail length distribution detected for the neoblast specific mRNAs *Smed-pcna* and *Smedwi-1* are only detected in X1 and X2 but the mRNAs are not detected in Xins FACS sorted populations. (E) No differences in poly(A) tail length distribution are detected for the tissue specific mRNA *Smed-mhc*, and only slight differences are detected in X1 and X2 but not in Xins fractions for the housekeeping mRNA *Smed-ef-2*.

As a further proof that the effects we observe are confined to stem cells we looked at gene expression, poly(A) tail length and the efficacy of *Smed-not1* knock down across FACS cellular compartments [51], [72]. Progressive increases in neoblast gene mRNA levels were confined to X1 and X2 populations of sorted cells from *Smed-not1(RNAi)* animals (Figure 8A, X1 and X2). Both of these sorted fractions contain neoblasts to different extents. Levels of these mRNAs were not increased in irradiation insensitive (Xins) sorted fractions from *Smed-not1(RNAi)* animals compared to controls (Figure 8A, Xins). This fraction contains primarily differentiated cells including CNS cells which also express *Smedtud-1* and *Smed-vasa-1*.

One possibility for the specificity we observe is that *Smed-not1* knock down has a high efficacy in stem cells but not in post-mitotic cells, as it has been suggested recently for *Smed-bruno-like* knock down [38], [73]. We performed qRT-PCR measurement of *Smed-not1* transcript levels and found that *Smed-not1* mRNA is consistently expressed in all cellular fractions (consistent with Figure S2 and Figure 1A) and that *Smed-not1* knock down significantly depletes *Smed-not1* in all sorted compartments compared to *control(RNAi)* animals (Figure 8B). Consequently the observed neoblast specificity is unlikely due to an absence of knock down in differentiated cells.

To finally link the mechanism of CCR4-NOT complex-mediated deadenylation to increased mRNA levels we also checked poly(A) tail lengths in FACS sorted cells. For *Smedtud-1*, *Smed-vasa-1*, *Smed-pcna* and *Smedwi-1* we observed a progressive increase in long poly(A) tails in the X1 compartment of stem cells (Figure 8C and D). This trend was also observed for these transcripts in X2 cells (Figure 8C and D). No poly(A) tail signal was detectable in Xins fractions for *Smed-pcna* and *Smedwi-1*, consistent with their low abundance in this fraction, and poly(A) tail length was not affected for *Smedtud-1* and *Smed-vasa-1* in Xins cells (Figure 8C and D). These data are in agreement with our irradiation based experiments (Figure 7). The poly(A) tail lengths of control mRNAs *Smed-ef-2* and *Smed-mhc* were only mildly or not affected by *Smed-not1* knock down (Figure 8E). These mild differences are likely due to CCR4-NOT mediated deadenylation, but again seem to be restricted to X1 and X2 cells, whereas Xins cells remain unaffected. To which extent all, most or only a subset of neoblast transcripts are affected after *Smed-not1* knock down remains an open question.

Taken together, these results clearly demonstrate that *Smed-not1* knock down induces a prominent increase of key transcripts expressed in neoblasts, that this accumulation occurs specifically in neoblasts, and that it is associated with an increased frequency of long poly(A) tails of these transcripts specifically in neoblasts. Furthermore, these results strongly suggest that the neoblast-specific increase of mRNA levels of genes such as *Smedtud-1*, *Smed-vasa-1* and *Smed-pcna* may be responsible for the impaired differentiation capacities of neoblasts observed in *Smed-not1(RNAi)* animals. It is likely that other genes expressed in neoblasts are similarly upregulated in *Smed-not1(RNAi)* animals and contribute to differentiation defects. Our results suggest a mechanism by which the differentiation capacities of neoblasts are dependent on CCR4-NOT mediated degradation of specific neoblast mRNAs.

## Discussion

Planarians are an emerging *in vivo* model for stem cell biology because of their unique stem cell population. In this study we used the planarian *S. mediterranea* as a model system to establish a function for the CCR4-NOT complex in stem cell regulation. We found that *Smed-not1* knock down abrogated regeneration and impaired homeostatic cell turnover. Interestingly, *Smed-not1* knock down primarily affects the stem cell compartment of *S. mediterranea* rather than inducing more widespread effects, even though the CCR4-NOT complex is the major deadenylating complex in eukaryotes and regulates at least 85% of mRNAs in yeast [74].

While we observed a stark and specific effect of *Smed-not1* knock down on deadenylation, it is still possible that other functions of the CCR4-NOT complex might also be impaired. The CCR4-NOT complex is involved in several steps of RNA metabolism [12], [13], [16] and further work is therefore needed to elucidate which ones are also at work in stem cells.

We observed effective gene knock down of *Smed-not1* even in differentiated cell fractions, but specific effects on neoblast transcripts were limited to stem cells. This fact suggests that targeted deadenylation by either RNA-binding proteins or miRNAs is providing specificity and is therefore central to stem cell differentiation and self-renewal properties. Consistently, several studies have implicated the CCR4-NOT complex in mRNA-specific deadenylation *via* targeted recruitment of the CCR4-NOT complex by RNA-binding proteins [26], [27], [30], [31], which are in turn known to be highly enriched and functionally important in neoblasts [43]–[46], [48]–[50], [52], [75]. It is possible to hypothesize that disruption of the CCR4-NOT complex via knock down of its scaffolding subunit might impair the protein-protein interactions needed to tether deadenylating activity to transcripts targeted by RNA-binding proteins or miRNAs for degradation, while general non-targeted deadenylation would remain relatively unaffected, therefore not causing a broader metabolic failure at earlier time points. In fact, the miRNA silencing complex protein GW182 specifically interacts with the Not1 subunit of the CCR4-NOT complex through specific and conserved domains [36], [37].

Despite the specificity of the effects seen in neoblast transcripts, mRNAs expressed elsewhere were also found to be affected. Both *Smed-agat-1* and, to a lesser extent, *Smed-nb.21.11e* were also affected. These results show that CCR4-NOT deadenylating activity is present in cell types other than neoblasts and that specificity is not due to restriction of activity to stem cells. Furthermore, the effects on well described neoblast progeny markers suggest that *Smed-not1* knock down likely influences several steps of cellular differentiation that may all contribute to the observed effects on homeostasis and regeneration.

The *Smed-not1* phenotype is progressive with respect to both the decreasing capacity of the animals to produce blastema cells and by the accumulation of mRNAs in stem cells. The phenotype results from a drop in neoblast progeny numbers, followed by stem cell loss. Similar neoblast and progeny dynamics have been shown by us in *Smedwi-2(RNAi)* organisms here and by another group in Smed-*CHD4(RNAi)* organisms [64]. Both *Smedwi-2* and *Smed-CHD4* knock downs initially affect neoblast differentiation rather than their self-renewal and proliferative capabilities [44], [64] which are only affected at later time points, similar to *Smed-not1* knock down. The ultimate cause for stem cell loss after the impairment of neoblast differentiation is unknown, and likely to be a broad failure in homeostasis as organs and tissues fail. However, for more than 15 days after *Smed-not1* dsRNA administration proliferating neoblasts are detectable in large numbers while regeneration is abrogated, suggesting neoblast loss is not a primary cause for the regeneration defect, instead implicating impaired neoblast differentiation capacities.

*Smed-not1* knock down induces an increased frequency of long poly(A) tails of *Smedtud-1*, *Smed-vasa-1*, *Smed-pcna* and *Smedwi-1* mRNAs as these same mRNAs accumulate. This regulation occurs specifically in neoblasts, rather than in the CNS, where two of these transcripts are also expressed. However, it is likely that *Smed-not1* knock down affects many more transcripts that contribute to failure in stem cell differentiation. Due to the lack of specific antibodies it is difficult to evaluate if the differences observed in transcript abundance and polyadenylation state affect the abundance of the proteins that these transcripts encode. However, taking into account that all four transcripts expressed in neoblasts analyzed accumulate in these stem cells, it is reasonable to conclude that *Smed-not1* knock down induces transcriptome-wide changes in stem cell expression patterns, and that these changes will likely affect protein levels.

Our results offer a new mechanistic insight into post-transcriptional regulation in neoblasts and its targets. After depletion of a post-transcriptional regulator many transcripts accumulate without being degraded, and this likely prevents neoblast differentiation, which needs the effective removal of these transcripts. The deadenylating activity of the CCR4-NOT complex is clearly central to this process. For genes like *Smedtud-1* and *Smed-vasa-1*, which are expressed in neoblasts and the CNS, we observe accumulation and increased frequency of long poly(A) tails of the transcript only in neoblasts. This suggests that the deadenylation of these mRNAs is regulated specifically during the onset of differentiation and requires the targeted recruitment of the CCR4-NOT complex by RNA-binding proteins, as has been described in other organisms [26], [27], [30], [31], [76]. Interestingly, several RNA binding proteins and post-transcriptional regulators have already been described as crucial for neoblast function [43], [44], [46], [48], and some of them have been already been functionally linked with the CCR4-NOT complex in other model organisms. Future research will help elucidate the mechanisms by which these proteins orchestrate planarian stem cell processes.

The CCR4-NOT complex has been shown to mediate the repressive function of both miRNAs and piRNAs [32]–[35]. Small RNAs are believed to be very important regulators of mammalian stem cells [77] and neoblasts [78], [79]. The *Smed-not1* RNAi phenotype is very similar to those of *Smedwi-2* and *Smedwi-3*, two Piwi proteins involved in piRNA regulation [44], [80]. Furthermore, several studies have highlighted the presence of miRNAs highly enriched in stem cells [78], [79], [81]. Future research will help in understanding if these phenotypic similarities reflect a functional link between Piwi proteins, piRNAs, miRNAs and the CCR4-NOT complex in planarian stem cells. Our results highlight the importance of the CCR4-NOT complex in the regulation of stem cells, the fact that post-transcriptional regulation of gene expression is a key element in the regulation of pluripotency, and that planarians will provide an excellent platform for these studies.

## Materials and Methods

### Organisms

Planarians of the asexual strain of *S. mediterranea* were kept and used as previously described [82].

### Sequences

The putative members of the *S. mediterranea* CCR4-NOT complex and other transcripts were identified in published *S. mediterranea* transcriptomic and genomic sequences [53], [58]–[60] and the longest transcripts for *Smed-not-1* confirmed by PCR and sequencing. The putative members of the *S. mediterranea* CCR4-NOT complex were identified by TBLASTN searches in the current assembly of the *S. mediterranea* genome and in the available transcriptomic data. In order to determine the number of loci for each of the components, the different transcripts identified were mapped to the *S. mediterranea* genome. The genomic sequence encoding *Smed-not1* was found split in two contigs (v31.001778 and v31.002774), the existence of one single transcript for these two genomic contigs was confirmed by PCR using the primers 5′-CATCGCAACAATGGAGAGAA-3′ and 5′-ATTTGAGCTGTATGGGCGAT-3′, each mapping to one of the two contigs. These PCR experiments revealed as well the existence of a 3 kb region not present in the *S. mediterranea* genomic data.

The full sequence of *Smed-not1* was obtained by *de novo* assembling the transcript from the raw transcriptomic data, using the known transcriptomic and genomic data as a guide. The *Smed-not1* sequence has been deposited in Genbank (accession KF781122).

The sequences of *Smedtud-1*, *Smed-vasa-1* (accession JQ425140) and *Smed-pcna* (accession EU856391) were found in our reference transcriptomic data, encoded by the transcripts AAA.454ESTABI.16133, AAA.454ESTABI.18605 and AAA.454ESTABI.22122 respectively. *Smed-ef-2* is encoded by the transcript AAA.454ESTABI.17328. The *Smedtud-1* sequence has been deposited in Genbank (accession KF781126).

### RNAi

RNAi experiments were performed as previously described [82]. *Control(RNAi)* worms were injected with dsRNA encoding for GFP, a gene not present in the *S. mediterranea* genome. dsRNA encoding for *Smed-not1* was prepared by in vitro transcription of a region of the *Smed-not1* gene. Briefly, an amplicon was generated from *S. mediterranea* reverse transcribed RNA with the primers 5′-GGCCGCGGTGTCCAAGAAAAAGCAAGTCAG-3′ and 5′-GCCCCGGCCAGCTGGCGTCAGTTTAGTGAA-3′, containing a 5′ adaptor sequence for the subsequent addition of T7 promoter. The product of this PCR was gel purified and subjected to another step of amplification with the primers 5′-GAGAATTCTAATACGACTCACTATAGGGCCGCGG-3′ and 5′-AGGGATCCTAATACGACTCACTATAGGCCCCGGC-3′ with the purpose of attaching T7 promoter sequences to both ends of the amplicon. The product of this PCR was further purified with Purelink DNA purification columns (Invitrogen) and used as a template for in vitro transcription using T7 RNA polymerase (Roche). The product of the in vitro transcription was treated with Turbo DNAse (Ambion), phenol extracted, precipitated in ethanol in presence of sodium acetate, glycogen and EDTA and re-suspended in water. dsRNA encoding GFP for use as a negative control was similarly prepared from a vector encoding the GFP gene. The final concentration of the injected solution was 1 µg/ µl. Animals were injected with a Nanoject II (Drummond) for three consecutive days and monitored or used for experiments in the subsequent days. Day 1 after RNAi is considered to be in all experiments the first day after the third dsRNA injection. Alive animals were imaged in a Zeiss Discovery V8 with a Zeiss AxioCam MRC camera.

### Irradiation

Irradiation was performed as previously described [79]. Animals were placed in a sealed γ-ray source and administered an irradiation dose of 100 Gy.

### *In situ* hybridization, immunohistochemistry and imaging

WMISH, FWMISH and IHC were performed and imaged as previously described [82]. When qualitative differences are shown, animals were processed and monitored in parallel. Riboprobes were generated by in vitro transcription of PCR products generated as described above, with only one T7 promoter linked to the 3′ end of the amplicon, and in the presence of digoxigenin-labelled UTP (Roche). The products of in vitro transcription reactions were then treated with Turbo DNAse (Ambion). Riboprobes were then precipitated in ethanol in the presence of LiCl and glycogen and resuspended in 50% formamide in TE buffer, 0.01% Tween.

The following primers were used:

*Smed-not1*: 5′- GGCCGCGGTGTCCAAGAAAAAGCAAGTCAG-3′

and 5′- GCCCCGGCCAGCTGGCGTCAGTTTAGTGAA-3′

*Smedtud-1*: 5′-GGCCGCGGCTAATGCCAGTTGACTGTCC-3′

and 5′-GCCCCGGCCCGAAAAAGTTCCGCATCACTT-3′

*Smedvas-1*: 5′-GGCCGCGGAGCTGTTGGAGTTGTTGGCTCAG-3′

and 5′-GCCCCGGCCCTAATCTTCGAGCCATTCAG-3′

*Smed-pcna*: 5′-GGCCGCGGATGGACTTGGATGGAGATCACT

*Smedwi-1*: 5′-GGCCGCGGAAGTGGTGGTATTCGAGAAGGA-3′

and 5′-GCCCCGGCCACGAATCGTAATCGGTTGTTCT-3′

*Smed-agat-1*: 5′-GGCCGCGGGAAATGATTGAGTCCACCATGA-3′

and 5′-GCCCCGGCCTGCAATATCTGGATAAGGAGCA-3′

*Smed-nb.21.11e*: 5′-GGCCGCGGGTGATTGCGTTCGCGTATATT-3′

and 5′-GCCCCGGCCATTTATCCAGCGCGTCATATTC-3′

Briefly, animals were killed in a 2%HCl solution, fixed in Carnoy's solution, bleached in a 8% H_2_O_2_/methanol, rehydrated, permeabilized with Proteinase K (Sigma), treated with 0.25% and 0.5% acetic anhydride in 0.1M triethanolamine pH 7.6, prehybridized and hybridized with digoxigenin labelled riboprobes (0.2 ng/µl, O/N at 56°C) They were then washed in buffers of increasing stringency, immunolabelled with anti-digoxigenin-alkaline-phosphatase antibody (Roche) and developed in the presence of NBT and BCIP (Roche). For FWMISH, an anti-digoxigenin-peroxidase antibody (Roche) was used and the signal was developed with the Tyramide Signal Amplification kit (Perkin Elmer). WMISH specimens were imaged on a Zeiss Discovery V8 equipped with a Zeiss AxioCam MRC camera. FWMISH specimens were imaged in a Leica SP3 confocal.

### Quantification of *Smed-agat-1*-positive cells

Animals (N = 8 per time point and treatment) were stained by FWMISH of *Smed-agat-1* and monitored by confocal microscopy. Cell counts were performed in z-projections of both the dorsal and ventral sides of the animals. 130 squares corresponding to 250 µm^2^ in both the dorsal and ventral parts of the animals were selected for counting along the length of the animals. Counts were performed using ImageJ software. Error bars represent standard error of the mean. Statistical significance was analyzed by Student's T test by comparing values from each sample to its respective control sample.

### Immunohistochemistry

Whole mount immunohistochemistry of phospho-histone-3 was performed as previously described [83]. Essentially, animals were fixed as above, blocked in a 1% BSA/PBS 0.3% triton X-100 solution, incubated overnight with anti-phospho-histone-3 (Millipore, 1/500 dilution) and anti-phospho-tyrosine (Cell Signaling, 1/200 dilution), washed and incubated in Alexa Fluor 488 and 568 secondary antibodies (Molecular Probes, 1/400 dilution). Animals were then washed, mounted in 70% glycerol/PBS and imaged with a Leica SP3 confocal and a Leica MZ16F fluorescence stereomicroscope and a Leica DFC 300Fx camera. Countings of phospho-histone-3 were performed with ImageJ software and normalized to the total area of the sample.

### qRT-PCR

qRT-PCR experiments were performed as previously described [48] with modifications. Essentially, total RNA from samples of 5 animals was extracted with Trizol reagent (Invitrogen) according to the manufacturer's instructions, and cDNAs were synthesized with SuperScriptIII Reverse Transcriptase (Invitrogen). qRT-PCR experiments were then performed using the Absolute qPCR SYBR Green Master Mix (Thermo Scientific). Experiments were performed on three biological replicates per time point and treatment. Each biological replicate was technically replicated three times in each reaction, each reaction was repeated three times. The gene *Smed-ef-2* was used for normalization.

The following primers were used:

*Smed-not1*: 5′-GACAGCGATTATGAACTGCC-3′ and 5′-CTGCTATGTTACTGGTGTTGAG-3′

*Smedwi-1*: 5′-AGTTCCTGTTCCAACGCATTATG-3′ and 5′-CTGGAGGAGTAACACCACGATGA-3′

*Smed-nb.21.11e*: 5′-GTCTCCCGCCAAATCAAGTA-3′ and 5′-TTTCATGCAATCTGCTTTCG-3′

*Smed-agat-1*:5′-TCCATCCAGAACCGATTGAT-3′ and 5′-CTCCCAAGTCATGGTGGACT-3′

*Smedtud-1*:5′-TGATGAAGGAACTTCGGGTGAT-3′ and 5′-TCTGAGCAACCGATTGAAACC-3′

*Smedvas-1*:5′-TGAAATGAACAAATCCCGAC-3′ and 5′-GAGAGCCAAACTAATTCCAG-3′

*Smed-pcna*:5′-GGCGCTTGGTAGTAATGATTCCCTA-3′ and 5′-TACCTAAGTGATCTCCATCCAAGTCC-3′

*Smed-ef-2*:5′-CAGCCAGTAGCTTTAAGCGATGA-3′ and 5′-ACTCTCAACGCTGCTGTCACTTC-3′

Statistical significance was measured by Student's T test by comparing values from each sample to their respective control sample.

### FACS

Fluorescence activated cell sorting (FACS) of planarian samples was performed as previously described [51], [72]. Analysis was performed with FlowJo.

### PAT assays

PAT assays were performed as previously described [69], [71] with minor modifications. A total of 400–1000 ng of total RNA extracted from five animals were used per each time point, replicate and treatment, except for FACS samples, in which 40 animals were used per time point and treatment and PAT reactions were performed with 100 ng of total RNA. Three biological replicates were analyzed per time point and treatment, and technically replicated at least twice, except for FACS samples, which were only technically replicated. *C. elegans* total RNA was spiked-in as a control and assayed with a primer for cpg-2.

RNA samples were incubated with 0.3 µg of 5′-phosphorylated oligo d(T) in a total volume of 8 µl, and heat denatured for 5 min at 65°C. Then, the following mixture was added: 4 µl of Super Script II First Strand Buffer, 0.5 µl of 0.1 M DTT, 2.25 µl of 10 mM ATP, 0.125 µl of RNAsin (Promega), 1.25 µl of 1 mM (each) dNTPs (Promega), and 1 µl of 2000 units/µl T4 DNA Ligase (New England Biolabs). The volume was then brought to a total of 20 µl with water, and the samples were incubated at 42°C for 30 min. in order to allow the saturation of polyA tails with oligo d(T). Then, 0.5 µl of an oligod(T)-anchor 100 µM was added (5′-GCGAGCTCCGCGGCCGCGTTTTTTTTTTTT-3′), and the mixture was incubated for 2 hours at 12°C to allow ligation of oligo d(T) molecules. Then, samples were pre-warmed at 42°C and 1 µl of SuperScript II (Invitrogen) was added. Finally the samples were incubated for 1 hour at 42°C and the SuperScript enzyme was heat inactivated at 70°C for 30 min.

These cDNAs were then used in PCR reactions, with fresh aliquots of the anchor primer and gene specific forward primers designed close to the 3′ region of the mRNA. The sequences of the mRNAs tested were obtained in our reference transcriptome (4). The following primers were used:

*Smedtud-1*: 5′-TGATGAAATAATGCTACCCGCGCAAT-3′

*Smed-vasa-1*: 5′-AGCCGACTTCTGAATGGCTCGAAGA-3′

*Smed-pcna*: 5′-CAAAGGCTGCACCTCTTTCTTCTCA-3′

*Smedwi-1*: 5′-CGTTGGCAAGATTCATCGTGGTGTT-3′

*Smed-agat-1*: 5′-TCGGATGTTAGAAGGCGAGGAGACC-3′

*Smed-nb.21.11e*: 5′- GACGGCCACTGTGACGCAGAAT-3′

*Smed-ef-2*: 5′-AACCCACTGGATCCCACAACGAAAC-3′

*Smed-eif-3*: 5′-GTTGCCCCATCGATTGGATACTTCG-3′

*Smed-mhc*: 5′- CGAGGAGCAAGTTCTGGACCTGGAA-3′

PCR reactions were carried away with DreamTaq (Fermentas) and amplified for 28–32 cycles of 94°C for 20 s., 65°C for 20 s. and 72 for 30 s. The products of the PCR reactions were analyzed on 1.5% agarose gels.

## Supporting Information

Figure S1 CCR4-NOT complex deadenylases do not induce a strong phenotype in planarians. (A) WMISH of *Smed-not6*, *Smed-not7A* and *Smed-not7B* in non irradiated and 3 days post irradiation animals. (B) *control(RNAi)*, *Smed-not6(RNAi)*, *Smed-not7A(RNAi)* and *Smed-not7B(RNAi)* animals cut 5 days after RNAi and monitored 6, 9 and 19 days after RNAi. Only a weak phenotype of delayed regeneration is observed for *Smed-not7A*. (C) WMISH of *Smed-H2B*, *Smed-nb.21.11e* and *Smed-agat-1* in *control(RNAi)*, *Smed-not6(RNAi)*, *Smed-not7A(RNAi)* and *Smed-not7B(RNAi)* animals 10 days after RNAi. No alteration of neoblast or progeny markers is observed.
(TIF)

Figure S2 *Smed-not1* is highly expressed across all planarian FACS sorted fractions. Expression levels, X1 vs Xins enrichment and corresponding gene IDs of the transcripts encoding for *Smedtud-1*, *Smed-vasa-1*, *Smed-pcna*, *Smed-ef2* and *Smed-not1*. Data taken from Onal *et al.* 2012. When transcripts are split into different transcriptomic sequences, all sequences are shown independently. The neoblast expressed transcripts *Smedtud-1*, *Smed-vasa-1* and *Smed-pcna* are most highly expressed in X1 fractions. The enrichment vs. Xins fractions is most high in *Smed-pcna* and lower in *Smed-vasa-1* and *Smedtud-1*, consistent with their expression in CNS. The enrichment vs. Xins fractions of *Smed-not1* is lower than all three neoblast expressed transcripts and more similar to the housekeeping gene *Smed-ef-2*. X1/Xins: log2(RPKM X1)-log2(RPKM Xins).
(TIF)

Figure S3 *Smed-not1* is required for planarian regeneration and homeostatic cell turnover. (A–F) *Control(RNAi)* (A) and *Smed-not1(RNAi)* animals cut 1 (B), 3 (C), 5 (D), 10 (E) and 15 (F) days after RNAi, and monitored every 2 days after transection. All panels are anterior wounds. Time of regeneration is indicated on top, total days after RNAi are indicated in each panel. Five animals were used per time point. 5 *control(RNAi)* animals were used for each of the time points, only one is shown (1 day) since no differences were detected among them. Crosses indicate death of all 5 animals. All *Smed-not1(RNAi)* animals are able to produce blastema cells, independent of the day of transection (B–F, day 4 of regeneration). However, the size of the blastema generated strongly depends on the day of transection. Animals cut earlier produce larger blastemas. Animals cut only 1 day after RNAi are able to regenerate photoreceptors (B, day 8 of regeneration) although later than *control(RNAi)* animals (A, day 6 of regeneration). All blastemas produced by *Smed-not1(RNAi)* animals eventually regress (B–F). (G–H) Intact *control(RNAi)* (G) and *Smed-not1(RNAi)* (H) animals 20 days after RNAi, anterior side is to the top. *Smed-not1* animals 20 days after RNAi display variable levels of head regression defects. Scale bars: 500 µm.
(TIF)

Figure S4 FACS analysis of planarian cell populations in *Smed-not1(RNAi)* animals. (A–B) FACS profiles of planarian cell populations in *Smed-not1(RNAi)* animals, *control(RNAi)* animals 10 and 15 days after RNAi (A) and animals 24 hours after irradiation (B). Planarian cells are dissociated and separated by FACS using a nuclear dye (Hoechst) and a cytoplasmic dye (Calcein). For RNAi animals, two biological replicates were technically replicated twice. Similarly, irradiated animals were technically replicated. Gating conditions to analyse percentage of X1 cells are indicated. *Smed-not1(RNAi)* animals show a mild but significant decrease in percentage of X1 cells (A, lower row), while irradiation almost completely eliminates X1 cells (B).
(TIF)

Figure S5 Dynamics of neoblasts and their progeny in *Smed-not1(RNAi)* and *Smedwi-2(RNAi)* animals. (A–F) WMISH of the neoblast marker *Smedwi-1* (A, D), the early neoblast progeny marker *Smed-nb.21.11e* (B, E) and the late neoblast progeny marker *Smed-agat-1* (C, F) in *control(RNAi)* animals (A–C) and *Smed-not1(RNAi)* animals (D–F) 20 days after RNAi. The level of *Smedwi-1* signals in *Smed-not1(RNAi)* animals is variable, including animals with almost normal expression (D, top panel) and animals with a prominent reduction in *Smedwi-1* levels (D, bottom panel). All *Smed-not1(RNAi)* animals present a severely reduced number of *Smed-nb.21.11e*-positive cells (E). The number of *Smed-agat-1*-positive cells is also variable (F), but all animals have reduced levels in the anterior part, typical behaviour of the marker *Smed-agat-1* upon neoblast perturbation. (G–R) WMISH of the neoblast marker *Smedwi-1* (G, J, M, P), the early neoblast progeny marker *Smed-nb.21.11e* (H, K, N, Q) and the late neoblast progeny marker *Smed-agat-1* (I, L, O, R) in *control(RNAi)* animals (G–I) and *Smedwi-2(RNAi)* animals 10 (J–L), 15 (M–O) and 20 (P–R) days after RNAi. *Smedwi-2(RNAi)* animals have detectable expression of *Smedwi-1* in almost all time points (J, M), although a severe decline in the level of *Smedwi-1* signals is detected 20 days after RNAi (P). The dynamics of progeny markers is also abnormal, with a progressive decline of *Smed-nb.21.11e* signals (N, Q) and of *Smed-agat-1* signals (L, O, R) that precedes the neoblast loss. Anterior is to the left. Scale bars: 500 µm.
(TIF)

Figure S6 Dynamics of stem cell transcripts and progeny transcripts after irradiation. (A–B) Quantification of the level of expression by qRT-PCR of the stem cell markers *Smedtud-1*, *Smedvas-1*, and *Smed-pcna* (A) and of the neoblast and progeny markers *Smedwi-1*, *Smed-nb.21.11e* and *Smed-agat-1* (B) in animals 1, 3 and 5 days after irradiation, normalized expression and relative to non irradiated samples. Error bars represent standard deviation. Animals 1 day after irradiation have around 10% of *Smed-pcna* transcripts of non-irradiated controls, and this number further decreases 3 and 5 days after irradiation. However, the expression of *Smedtud-1* and *Smedvas-1* mRNAs only decreases to around 70% and 40% respectively of the level of non irradiated controls, reflecting expression that does not localize to neoblasts and is therefore not eliminated by irradiation. Similar to *Smed-pcna*, the level of *Smedwi-1* transcripts decreases to around 10% of the expression in non irradiated controls and becomes almost undetectable later. The levels of the progeny specific mRNAs *Smed-nb.21.11e* and *Smed-agat-1* decrease progressively at later time points of irradiation. Therefore, around 90% of the neoblast specific transcripts are eliminated only 1 day after irradiation while most of the expression of progeny specific transcripts is still detected and the non-neoblast expression of *Smedtud-1* and *Smedvas-1* localized in the CNS is not eliminated by irradiation.
(TIF)

Table S1 *In silico* search of CCR4-NOT complex components in *S. mediterranea*. Summary of the CCR4-NOT complex components found *in silico* in *S. mediterranea*. Each of the described components of the yeast, *Drosophila melanogaster* and human CCR4-NOT complexes is indicated. The column “*S. mediterranea*” indicates the given name for each of the genes, the column “Contig Smed genome” indicates the genomic contigs in which each locus was found, followed by the transcriptomic datasets from Blythe *et al.* and Adamidi *et al.* The column “*D. japonica*” indicates the accession numbers of the CCR4-NOT components previously described in this planarian species. Nine different components of the CCR4-NOT complex were found both in genomic and transcriptomic sequences, corresponding to the orthologues of all metazoan CCR4-NOT complex components. Similar to humans, two paralogues of the not7/caf1 gene were found (*Smed-not7A* and *Smed-not7B*) in both genomic and transcriptomic sequences and two additional genomic loci encoding two similar versions of an additional not7/caf1 gene were found (*Smed-not7C.1* and *Smed-not7C.2*). However, the transcripts encoded by these two genomic loci were not found in transcriptomic datasets, and therefore they are possible pseudogenes. No orthologue of the yeast specific not5 was found, but one orthologue of the metazoan specific not10 was found (*Smed-not10*). We found several transcripts mapping to the same genomic locus for most of the genes, encoding different regions of the gene or different splicing variants. The *Smed-not1* gene was split in two different contigs (v31.001778 and v31.002774) encoding respectively the 5′ and 3′ regions of the same gene. PCR experiments confirmed that they correspond to the same transcript.
(PDF)

## References

[1] Seydoux G, Braun RE (2006) Pathway to totipotency: lessons from germ cells. Cell 127: 891–904. 17129777 10.1016/j.cell.2006.11.016

[2] Garneau NL, Wilusz J, Wilusz CJ (2007) The highways and byways of mRNA decay. Nat Rev Mol Cell Biol 8: 113–126. 17245413 10.1038/nrm2104

[3] Balagopal V, Fluch L, Nissan T (2012) Ways and means of eukaryotic mRNA decay. Biochim Biophys Acta 1819: 593–603. 22266130 10.1016/j.bbagrm.2012.01.001

[4] Wu X, Brewer G (2012) The regulation of mRNA stability in mammalian cells: 2.0. Gene 500: 10–21. 22452843 10.1016/j.gene.2012.03.021 PMC3340483

[5] Wang Y, Liu CL, Storey JD, Tibshirani RJ, Herschlag D, et al (2002) Precision and functional specificity in mRNA decay. Proc Natl Acad Sci U S A 99: 5860–5865. 11972065 10.1073/pnas.092538799 PMC122867

[6] Munchel SE, Shultzaberger RK, Takizawa N, Weis K (2011) Dynamic profiling of mRNA turnover reveals gene-specific and system-wide regulation of mRNA decay. Mol Biol Cell 22: 2787–2795. 21680716 10.1091/mbc.E11-01-0028 PMC3145553

[7] Glisovic T, Bachorik JL, Yong J, Dreyfuss G (2008) RNA-binding proteins and post-transcriptional gene regulation. FEBS Lett 582: 1977–1986. 18342629 10.1016/j.febslet.2008.03.004 PMC2858862

[8] Wiederhold K, Passmore LA (2010) Cytoplasmic deadenylation: regulation of mRNA fate. Biochem Soc Trans 38: 1531–1536. 21118121 10.1042/BST0381531 PMC3890232

[9] Zhang X, Virtanen A, Kleiman FE (2010) To polyadenylate or to deadenylate: that is the question. Cell Cycle 9: 4437–4449. 21084869 10.4161/cc.9.22.13887 PMC3048043

[10] Weill L, Belloc E, Bava FA, Mendez R (2012) Translational control by changes in poly(A) tail length: recycling mRNAs. Nat Struct Mol Biol 19: 577–585. 22664985 10.1038/nsmb.2311

[11] Collart MA, Panasenko OO (2011) The Ccr4-Not complex. Gene 10.1016/j.gene.2011.09.033 22027279

[12] Miller JE, Reese JC (2012) Ccr4-Not complex: the control freak of eukaryotic cells. Crit Rev Biochem Mol Biol 47: 315–333. 22416820 10.3109/10409238.2012.667214 PMC3376659

[13] Wahle E, Winkler GS (2013) RNA decay machines: deadenylation by the Ccr4-not and Pan2-Pan3 complexes. Biochim Biophys Acta 1829: 561–570. 23337855 10.1016/j.bbagrm.2013.01.003

[14] Kerr SC, Azzouz N, Fuchs SM, Collart MA, Strahl BD, et al (2011) The Ccr4-Not complex interacts with the mRNA export machinery. PLoS One 6: e18302. 21464899 10.1371/journal.pone.0018302 PMC3065485

[15] Panasenko O, Landrieux E, Feuermann M, Finka A, Paquet N, et al (2006) The yeast Ccr4-Not complex controls ubiquitination of the nascent-associated polypeptide (NAC-EGD) complex. J Biol Chem 281: 31389–31398. 16926149 10.1074/jbc.M604986200

[16] Kruk JA, Dutta A, Fu J, Gilmour DS, Reese JC (2011) The multifunctional Ccr4-Not complex directly promotes transcription elongation. Genes Dev 25: 581–593. 21406554 10.1101/gad.2020911 PMC3059832

[17] Reese JC (2013) The control of elongation by the yeast Ccr4-not complex. Biochim Biophys Acta 1829: 127–133. 22975735 10.1016/j.bbagrm.2012.09.001 PMC3545033

[18] Collart MA, Panasenko OO, Nikolaev SI (2013) The Not3/5 subunit of the Ccr4-Not complex: a central regulator of gene expression that integrates signals between the cytoplasm and the nucleus in eukaryotic cells. Cell Signal 25: 743–751. 23280189 10.1016/j.cellsig.2012.12.018

[19] Maillet L, Tu C, Hong YK, Shuster EO, Collart MA (2000) The essential function of Not1 lies within the Ccr4-Not complex. J Mol Biol 303: 131–143. 11023781 10.1006/jmbi.2000.4131

[20] Albert TK, Lemaire M, van Berkum NL, Gentz R, Collart MA, et al (2000) Isolation and characterization of human orthologs of yeast CCR4-NOT complex subunits. Nucleic Acids Res 28: 809–817. 10637334 10.1093/nar/28.3.809 PMC102560

[21] Temme C, Zaessinger S, Meyer S, Simonelig M, Wahle E (2004) A complex containing the CCR4 and CAF1 proteins is involved in mRNA deadenylation in Drosophila. EMBO J 23: 2862–2871. 15215893 10.1038/sj.emboj.7600273 PMC514940

[22] Nousch M, Techritz N, Hampel D, Millonigg S, Eckmann CR (2013) The Ccr4-Not deadenylase complex constitutes the major poly(A) removal activity in C. elegans. J Cell Sci 126 (Pt 18) 4274–85. 23843623 10.1242/jcs.132936

[23] Lau NC, Kolkman A, van Schaik FM, Mulder KW, Pijnappel WW, et al (2009) Human Ccr4-Not complexes contain variable deadenylase subunits. Biochem J 422: 443–453. 19558367 10.1042/BJ20090500

[24] Aslam A, Mittal S, Koch F, Andrau JC, Winkler GS (2009) The Ccr4-NOT deadenylase subunits CNOT7 and CNOT8 have overlapping roles and modulate cell proliferation. Mol Biol Cell 20: 3840–3850. 19605561 10.1091/mbc.E09-02-0146 PMC2735483

[25] Mittal S, Aslam A, Doidge R, Medica R, Winkler GS (2011) The Ccr4a (CNOT6) and Ccr4b (CNOT6L) deadenylase subunits of the human Ccr4-Not complex contribute to the prevention of cell death and senescence. Mol Biol Cell 22: 748–758. 21233283 10.1091/mbc.E10-11-0898 PMC3057700

[26] Kadyrova LY, Habara Y, Lee TH, Wharton RP (2007) Translational control of maternal Cyclin B mRNA by Nanos in the Drosophila germline. Development 134: 1519–1527. 17360772 10.1242/dev.002212

[27] Suzuki A, Igarashi K, Aisaki K, Kanno J, Saga Y (2010) NANOS2 interacts with the CCR4-NOT deadenylation complex and leads to suppression of specific RNAs. Proc Natl Acad Sci U S A 107: 3594–3599. 20133598 10.1073/pnas.0908664107 PMC2840499

[28] Van Etten J, Schagat TL, Hrit J, Weidmann CA, Brumbaugh J, et al (2012) Human Pumilio proteins recruit multiple deadenylases to efficiently repress messenger RNAs. J Biol Chem 287: 36370–36383. 22955276 10.1074/jbc.M112.373522 PMC3476303

[29] Goldstrohm AC, Wickens M (2008) Multifunctional deadenylase complexes diversify mRNA control. Nat Rev Mol Cell Biol 9: 337–344. 18334997 10.1038/nrm2370

[30] Zaessinger S, Busseau I, Simonelig M (2006) Oskar allows nanos mRNA translation in Drosophila embryos by preventing its deadenylation by Smaug/CCR4. Development 133: 4573–4583. 17050620 10.1242/dev.02649

[31] Chicoine J, Benoit P, Gamberi C, Paliouras M, Simonelig M, et al (2007) Bicaudal-C recruits CCR4-NOT deadenylase to target mRNAs and regulates oogenesis, cytoskeletal organization, and its own expression. Dev Cell 13: 691–704. 17981137 10.1016/j.devcel.2007.10.002

[32] Behm-Ansmant I, Rehwinkel J, Doerks T, Stark A, Bork P, et al (2006) mRNA degradation by miRNAs and GW182 requires both CCR4:NOT deadenylase and DCP1:DCP2 decapping complexes. Genes Dev 20: 1885–1898. 16815998 10.1101/gad.1424106 PMC1522082

[33] Eulalio A, Huntzinger E, Nishihara T, Rehwinkel J, Fauser M, et al (2009) Deadenylation is a widespread effect of miRNA regulation. RNA 15: 21–32. 19029310 10.1261/rna.1399509 PMC2612776

[34] Fabian MR, Mathonnet G, Sundermeier T, Mathys H, Zipprich JT, et al (2009) Mammalian miRNA RISC recruits CAF1 and PABP to affect PABP-dependent deadenylation. Mol Cell 35: 868–880. 19716330 10.1016/j.molcel.2009.08.004 PMC2803087

[35] Rouget C, Papin C, Boureux A, Meunier AC, Franco B, et al (2010) Maternal mRNA deadenylation and decay by the piRNA pathway in the early Drosophila embryo. Nature 467: 1128–1132. 20953170 10.1038/nature09465 PMC4505748

[36] Fabian MR, Cieplak MK, Frank F, Morita M, Green J, et al (2011) miRNA-mediated deadenylation is orchestrated by GW182 through two conserved motifs that interact with CCR4-NOT. Nat Struct Mol Biol 18: 1211–1217. 21984185 10.1038/nsmb.2149

[37] Chekulaeva M, Mathys H, Zipprich JT, Attig J, Colic M, et al (2011) miRNA repression involves GW182-mediated recruitment of CCR4-NOT through conserved W-containing motifs. Nat Struct Mol Biol 18: 1218–1226. 21984184 10.1038/nsmb.2166 PMC3885283

[38] Zheng X, Dumitru R, Lackford BL, Freudenberg JM, Singh AP, et al (2012) Cnot1, Cnot2, and Cnot3 maintain mouse and human ESC identity and inhibit extraembryonic differentiation. Stem Cells 30: 910–922. 22367759 10.1002/stem.1070 PMC3787717

[39] Aboobaker AA (2011) Planarian stem cells: a simple paradigm for regeneration. Trends Cell Biol 21: 304–311. 21353778 10.1016/j.tcb.2011.01.005

[40] Gentile L, Cebria F, Bartscherer K (2011) The planarian flatworm: an in vivo model for stem cell biology and nervous system regeneration. Dis Model Mech 4: 12–19. 21135057 10.1242/dmm.006692 PMC3014342

[41] Rink JC (2013) Stem cell systems and regeneration in planaria. Dev Genes Evol 223: 67–84. 23138344 10.1007/s00427-012-0426-4 PMC3552358

[42] Wagner DE, Wang IE, Reddien PW (2011) Clonogenic neoblasts are pluripotent adult stem cells that underlie planarian regeneration. Science 332: 811–816. 21566185 10.1126/science.1203983 PMC3338249

[43] Guo T, Peters AH, Newmark PA (2006) A Bruno-like gene is required for stem cell maintenance in planarians. Dev Cell 11: 159–169. 16890156 10.1016/j.devcel.2006.06.004

[44] Reddien PW, Oviedo NJ, Jennings JR, Jenkin JC, Sanchez Alvarado A (2005) SMEDWI-2 is a PIWI-like protein that regulates planarian stem cells. Science 310: 1327–1330. 16311336 10.1126/science.1116110

[45] Rouhana L, Shibata N, Nishimura O, Agata K (2010) Different requirements for conserved post-transcriptional regulators in planarian regeneration and stem cell maintenance. Dev Biol 341: 429–443. 20230812 10.1016/j.ydbio.2010.02.037

[46] Salvetti A, Rossi L, Lena A, Batistoni R, Deri P, et al (2005) DjPum, a homologue of Drosophila Pumilio, is essential to planarian stem cell maintenance. Development 132: 1863–1874. 15772127 10.1242/dev.01785

[47] Shibata N, Rouhana L, Agata K (2010) Cellular and molecular dissection of pluripotent adult somatic stem cells in planarians. Dev Growth Differ 52: 27–41. 20078652 10.1111/j.1440-169X.2009.01155.x

[48] Solana J, Lasko P, Romero R (2009) Spoltud-1 is a chromatoid body component required for planarian long-term stem cell self-renewal. Dev Biol 328: 410–421. 19389344 10.1016/j.ydbio.2009.01.043 PMC2674143

[49] Rouhana L, Vieira AP, Roberts-Galbraith RH, Newmark PA (2012) PRMT5 and the role of symmetrical dimethylarginine in chromatoid bodies of planarian stem cells. Development 139: 1083–1094. 22318224 10.1242/dev.076182 PMC3283120

[50] Solana J, Kao D, Mihaylova Y, Jaber-Hijazi F, Malla S, et al (2012) Defining the molecular profile of planarian pluripotent stem cells using a combinatorial RNAseq, RNA interference and irradiation approach. Genome Biol 13: R19. 22439894 10.1186/gb-2012-13-3-r19 PMC3439970

[51] Onal P, Grun D, Adamidi C, Rybak A, Solana J, et al (2012) Gene expression of pluripotency determinants is conserved between mammalian and planarian stem cells. EMBO J 31: 2755–2769. 22543868 10.1038/emboj.2012.110 PMC3380209

[52] Labbe RM, Irimia M, Currie KW, Lin A, Zhu SJ, et al (2012) A comparative transcriptomic analysis reveals conserved features of stem cell pluripotency in planarians and mammals. Stem Cells 30: 1734–1745. 22696458 10.1002/stem.1144 PMC4161212

[53] Blythe MJ, Kao D, Malla S, Rowsell J, Wilson R, et al (2010) A dual platform approach to transcript discovery for the planarian Schmidtea mediterranea to establish RNAseq for stem cell and regeneration biology. PLoS One 5: e15617. 21179477 10.1371/journal.pone.0015617 PMC3001875

[54] Wagner DE, Ho JJ, Reddien PW (2012) Genetic regulators of a pluripotent adult stem cell system in planarians identified by RNAi and clonal analysis. Cell Stem Cell 10: 299–311. 22385657 10.1016/j.stem.2012.01.016 PMC3338251

[55] Solana J (2013) Closing the circle of germline and stem cells: the Primordial Stem Cell hypothesis. Evodevo 4: 2. 23294912 10.1186/2041-9139-4-2 PMC3599645

[56] Aboobaker AA, Kao D (2012) A lack of commitment for over 500 million years: conserved animal stem cell pluripotency. EMBO J 31: 2747–2749. 22562151 10.1038/emboj.2012.131 PMC3380215

[57] Jaber-Hijazi F, Lo PJ, Mihaylova Y, Foster JM, Benner JS, et al (2013) Planarian MBD2/3 is required for adult stem cell pluripotency independently of DNA methylation. Developmental biology 384: 141–153. 24063805 10.1016/j.ydbio.2013.09.020 PMC3824064

[58] Abril JF, Cebria F, Rodriguez-Esteban G, Horn T, Fraguas S, et al (2010) Smed454 dataset: unravelling the transcriptome of Schmidtea mediterranea. BMC Genomics 11: 731. 21194483 10.1186/1471-2164-11-731 PMC3022928

[59] Adamidi C, Wang Y, Gruen D, Mastrobuoni G, You X, et al (2011) De novo assembly and validation of planaria transcriptome by massive parallel sequencing and shotgun proteomics. Genome Res 21: 1193–1200. 21536722 10.1101/gr.113779.110 PMC3129261

[60] Robb SM, Ross E, Sanchez Alvarado A (2008) SmedGD: the Schmidtea mediterranea genome database. Nucleic Acids Res 36: D599–606. 17881371 10.1093/nar/gkm684 PMC2238899

[61] Mochizuki K, Nishimiya-Fujisawa C, Fujisawa T (2001) Universal occurrence of the vasa-related genes among metazoans and their germline expression in Hydra. Dev Genes Evol 211: 299–308. 11466525 10.1007/s004270100156

[62] Orii H, Sakurai T, Watanabe K (2005) Distribution of the stem cells (neoblasts) in the planarian Dugesia japonica. Dev Genes Evol 215: 143–157. 15657737 10.1007/s00427-004-0460-y

[63] Inoue T, Kumamoto H, Okamoto K, Umesono Y, Sakai M, et al (2004) Morphological and functional recovery of the planarian photosensing system during head regeneration. Zoolog Sci 21: 275–283. 15056922 10.2108/zsj.21.275

[64] Reddien PW, Bermange AL, Murfitt KJ, Jennings JR, Sanchez Alvarado A (2005) Identification of genes needed for regeneration, stem cell function, and tissue homeostasis by systematic gene perturbation in planaria. Dev Cell 8: 635–649. 15866156 10.1016/j.devcel.2005.02.014 PMC2267917

[65] Scimone ML, Meisel J, Reddien PW (2010) The Mi-2-like Smed-CHD4 gene is required for stem cell differentiation in the planarian Schmidtea mediterranea. Development 137: 1231–1241. 20223763 10.1242/dev.042051 PMC2847463

[66] Eisenhoffer GT, Kang H, Sanchez Alvarado A (2008) Molecular analysis of stem cells and their descendants during cell turnover and regeneration in the planarian Schmidtea mediterranea. Cell Stem Cell 3: 327–339. 18786419 10.1016/j.stem.2008.07.002 PMC2614339

[67] Pearson BJ, Sanchez Alvarado A (2010) A planarian p53 homolog regulates proliferation and self-renewal in adult stem cell lineages. Development 137: 213–221. 20040488 10.1242/dev.044297 PMC2799157

[68] Gamberi C, Gottlieb E (2002) Internally controlled poly(A) tail assay to study gene regulation. Biotechniques 33: 476–480, 476, 478, 480. 12238753 10.2144/02333bm02

[69] Gamberi C, Peterson DS, He L, Gottlieb E (2002) An anterior function for the Drosophila posterior determinant Pumilio. Development 129: 2699–2710. 12015297 10.1242/dev.129.11.2699

[70] Salles FJ, Lieberfarb ME, Wreden C, Gergen JP, Strickland S (1994) Coordinate initiation of Drosophila development by regulated polyadenylation of maternal messenger RNAs. Science 266: 1996–1999. 7801127 10.1126/science.7801127

[71] Salles FJ, Strickland S (1995) Rapid and sensitive analysis of mRNA polyadenylation states by PCR. PCR Methods Appl 4: 317–321. 7580923 10.1101/gr.4.6.317

[72] Hayashi T, Asami M, Higuchi S, Shibata N, Agata K (2006) Isolation of planarian X-ray-sensitive stem cells by fluorescence-activated cell sorting. Dev Growth Differ 48: 371–380. 16872450 10.1111/j.1440-169X.2006.00876.x

[73] Rouhana L, Weiss JA, Forsthoefel DJ, Lee H, King RS, et al (2013) RNA interference by feeding in vitro-synthesized double-stranded RNA to planarians: methodology and dynamics. Dev Dyn 242: 718–730. 23441014 10.1002/dvdy.23950 PMC3909682

[74] Azzouz N, Panasenko OO, Deluen C, Hsieh J, Theiler G, et al (2009) Specific roles for the Ccr4-Not complex subunits in expression of the genome. RNA 15: 377–383. 19155328 10.1261/rna.1348209 PMC2657018

[75] Resch AM, Palakodeti D, Lu YC, Horowitz M, Graveley BR (2012) Transcriptome analysis reveals strain-specific and conserved stemness genes in Schmidtea mediterranea. PLoS One 7: e34447. 22496805 10.1371/journal.pone.0034447 PMC3319590

[76] Goldstrohm AC, Seay DJ, Hook BA, Wickens M (2007) PUF protein-mediated deadenylation is catalyzed by Ccr4p. J Biol Chem 282: 109–114. 17090538 10.1074/jbc.M609413200

[77] Marson A, Levine SS, Cole MF, Frampton GM, Brambrink T, et al (2008) Connecting microRNA genes to the core transcriptional regulatory circuitry of embryonic stem cells. Cell 134: 521–533. 18692474 10.1016/j.cell.2008.07.020 PMC2586071

[78] Friedlander MR, Adamidi C, Han T, Lebedeva S, Isenbarger TA, et al (2009) High-resolution profiling and discovery of planarian small RNAs. Proc Natl Acad Sci U S A 106: 11546–11551. 19564616 10.1073/pnas.0905222106 PMC2703670

[79] Gonzalez-Estevez C, Arseni V, Thambyrajah RS, Felix DA, Aboobaker AA (2009) Diverse miRNA spatial expression patterns suggest important roles in homeostasis and regeneration in planarians. Int J Dev Biol 53: 493–505. 19247960 10.1387/ijdb.082825cg

[80] Palakodeti D, Smielewska M, Lu YC, Yeo GW, Graveley BR (2008) The PIWI proteins SMEDWI-2 and SMEDWI-3 are required for stem cell function and piRNA expression in planarians. RNA 14: 1174–1186. 18456843 10.1261/rna.1085008 PMC2390803

[81] Lu YC, Smielewska M, Palakodeti D, Lovci MT, Aigner S, et al (2009) Deep sequencing identifies new and regulated microRNAs in Schmidtea mediterranea. RNA 15: 1483–1491. 19553344 10.1261/rna.1702009 PMC2714757

[82] Felix DA, Aboobaker AA (2010) The TALE class homeobox gene Smed-prep defines the anterior compartment for head regeneration. PLoS Genet 6: e1000915. 20422023 10.1371/journal.pgen.1000915 PMC2858555

[83] Cebria F, Newmark PA (2005) Planarian homologs of netrin and netrin receptor are required for proper regeneration of the central nervous system and the maintenance of nervous system architecture. Development 132: 3691–3703. 16033796 10.1242/dev.01941

