# Supplementary figures and images for: The CCR4-NOT Complex Mediates Deadenylation and Degradation of Stem Cell mRNAs and Promotes Planarian Stem Cell Differentiation
Source: PLoS Genet. 2013 Dec 19;9(12):e1004003. doi: 10.1371/journal.pgen.1004003 (PMC3868585; doi:10.1371/journal.pgen.1004003)

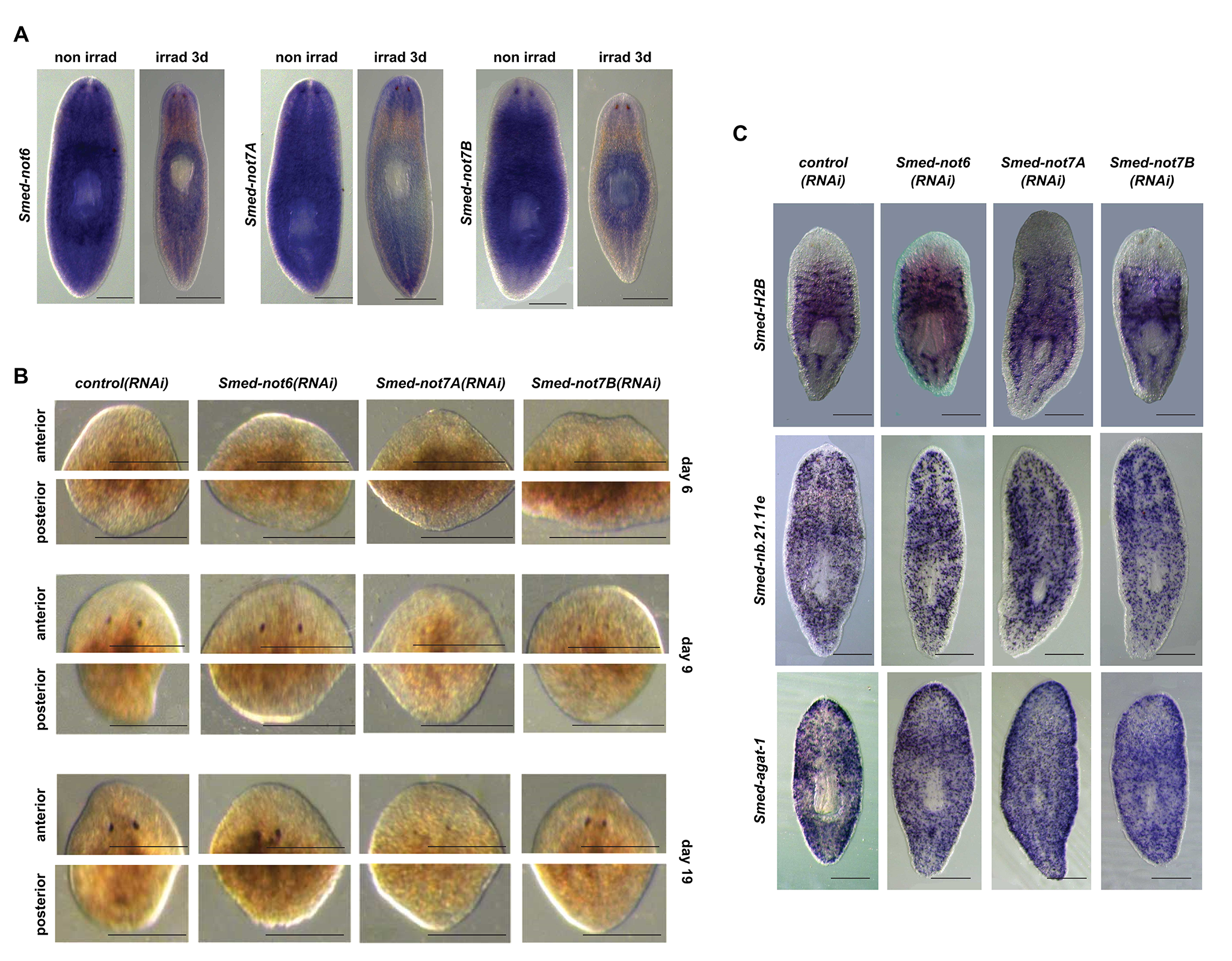

Supplement: Figure S1 — CCR4-NOT complex deadenylases do not induce a strong phenotype in planarians. (A) WMISH of Smed-not6, Smed-not7A and Smed-not7B in non irradiated and 3 days post irradiation animals. (B) control(RNAi), Smed-not6(RNAi), Smed-not7A(RNAi) and Smed-not7B(RNAi) animals cut 5 days after RNAi and monitored 6, 9 and 19 days after RNAi. Only a weak phenotype of delayed regeneration is observed for Smed-not7A. (C) WMISH of Smed-H2B, Smed-nb.21.11e and Smed-agat-1 in control(RNAi), Smed-not6(RNAi), Smed-not7A(RNAi) and Smed-not7B(RNAi) animals 10 days after RNAi. No alteration of neoblast or progeny markers is observed. (TIF) [file pgen.1004003.s001.tif]

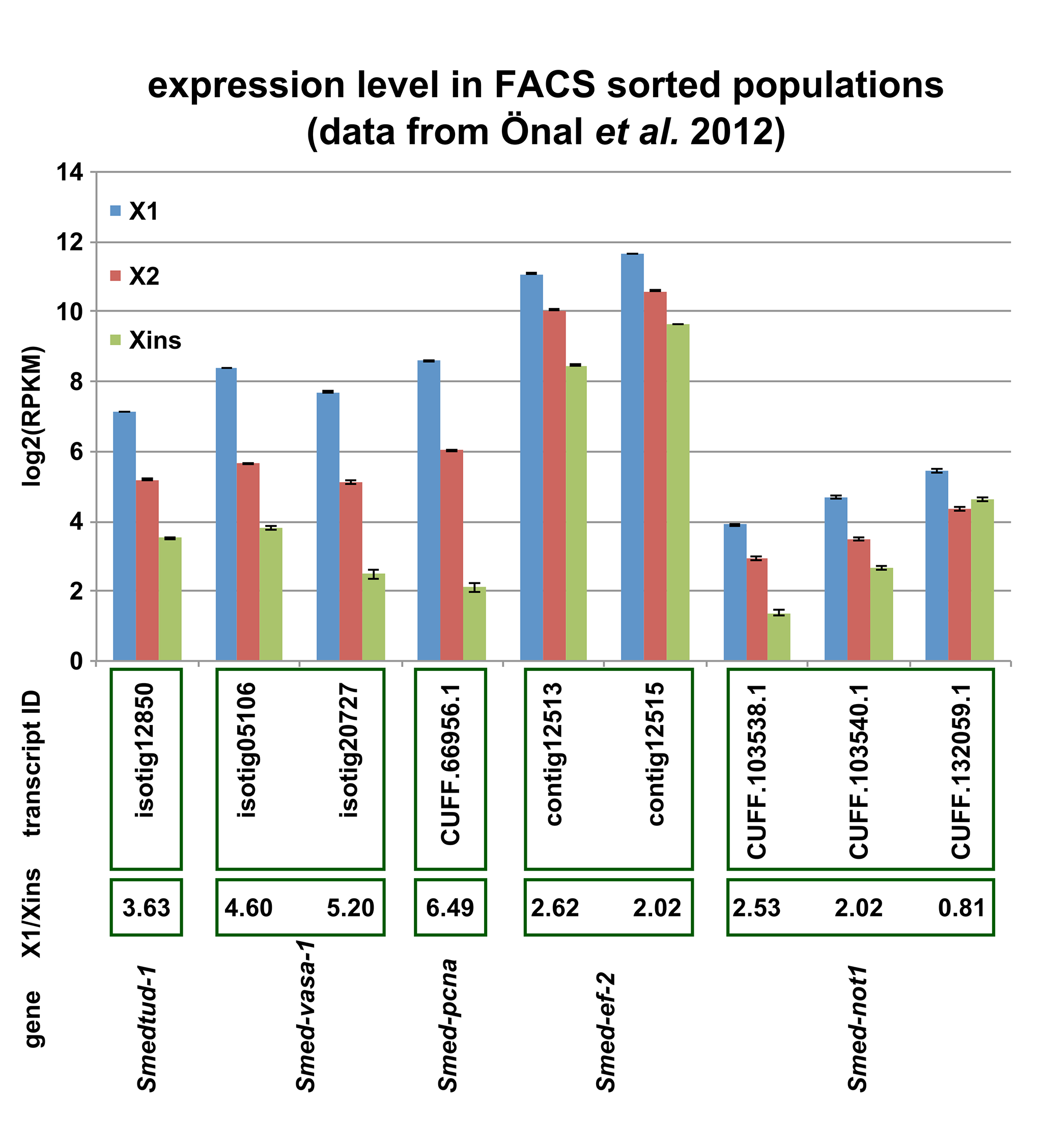

Supplement: Figure S2 — Smed-not1 is highly expressed across all planarian FACS sorted fractions. Expression levels, X1 vs Xins enrichment and corresponding gene IDs of the transcripts encoding for Smedtud-1, Smed-vasa-1, Smed-pcna, Smed-ef2 and Smed-not1. Data taken from Onal et al. 2012. When transcripts are split into different transcriptomic sequences, all sequences are shown independently. The neoblast expressed transcripts Smedtud-1, Smed-vasa-1 and Smed-pcna are most highly expressed in X1 fractions. The enrichment vs. Xins fractions is most high in Smed-pcna and lower in Smed-vasa-1 and Smedtud-1, consistent with their expression in CNS. The enrichment vs. Xins fractions of Smed-not1 is lower than all three neoblast expressed transcripts and more similar to the housekeeping gene Smed-ef-2. X1/Xins: log2(RPKM X1)-log2(RPKM Xins). (TIF) [file pgen.1004003.s002.tif]

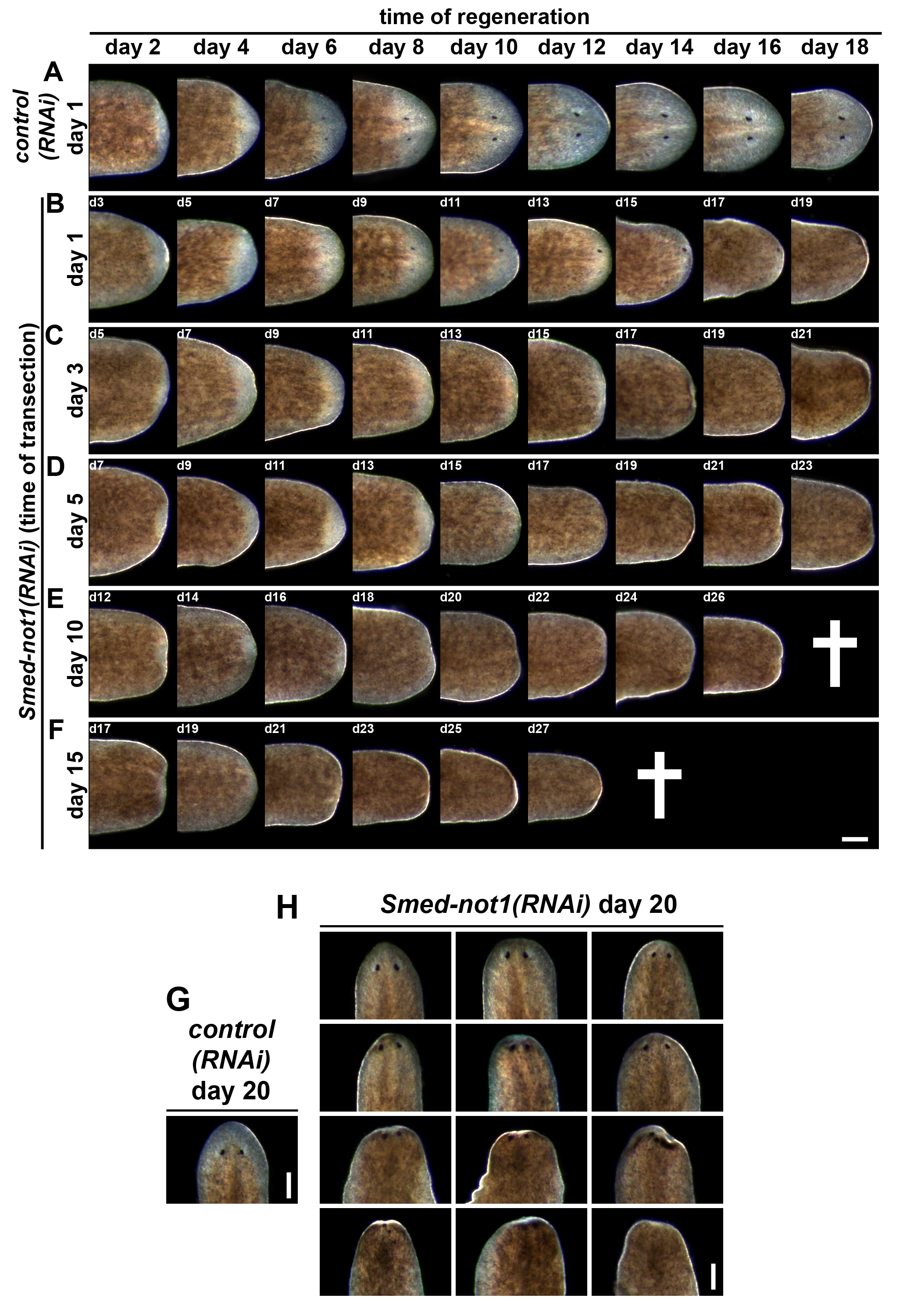

Supplement: Figure S3 — Smed-not1 is required for planarian regeneration and homeostatic cell turnover. (A–F) Control(RNAi) (A) and Smed-not1(RNAi) animals cut 1 (B), 3 (C), 5 (D), 10 (E) and 15 (F) days after RNAi, and monitored every 2 days after transection. All panels are anterior wounds. Time of regeneration is indicated on top, total days after RNAi are indicated in each panel. Five animals were used per time point. 5 control(RNAi) animals were used for each of the time points, only one is shown (1 day) since no differences were detected among them. Crosses indicate death of all 5 animals. All Smed-not1(RNAi) animals are able to produce blastema cells, independent of the day of transection (B–F, day 4 of regeneration). However, the size of the blastema generated strongly depends on the day of transection. Animals cut earlier produce larger blastemas. Animals cut only 1 day after RNAi are able to regenerate photoreceptors (B, day 8 of regeneration) although later than control(RNAi) animals (A, day 6 of regeneration). All blastemas produced by Smed-not1(RNAi) animals eventually regress (B–F). (G–H) Intact control(RNAi) (G) and Smed-not1(RNAi) (H) animals 20 days after RNAi, anterior side is to the top. Smed-not1 animals 20 days after RNAi display variable levels of head regression defects. Scale bars: 500 µm. (TIF) [file pgen.1004003.s003.tif]

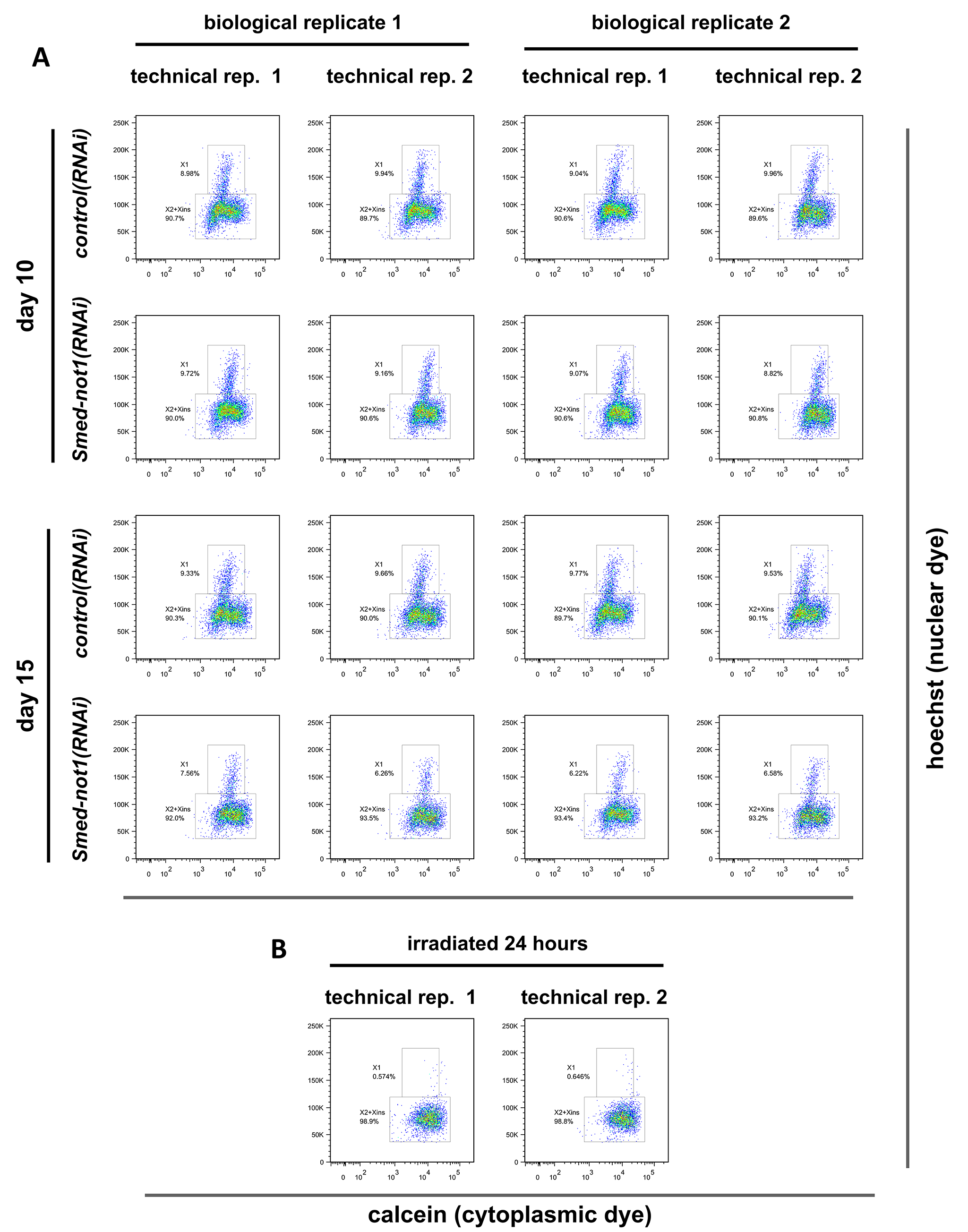

Supplement: Figure S4 — FACS analysis of planarian cell populations in Smed-not1(RNAi) animals. (A–B) FACS profiles of planarian cell populations in Smed-not1(RNAi) animals, control(RNAi) animals 10 and 15 days after RNAi (A) and animals 24 hours after irradiation (B). Planarian cells are dissociated and separated by FACS using a nuclear dye (Hoechst) and a cytoplasmic dye (Calcein). For RNAi animals, two biological replicates were technically replicated twice. Similarly, irradiated animals were technically replicated. Gating conditions to analyse percentage of X1 cells are indicated. Smed-not1(RNAi) animals show a mild but significant decrease in percentage of X1 cells (A, lower row), while irradiation almost completely eliminates X1 cells (B). (TIF) [file pgen.1004003.s004.tif]

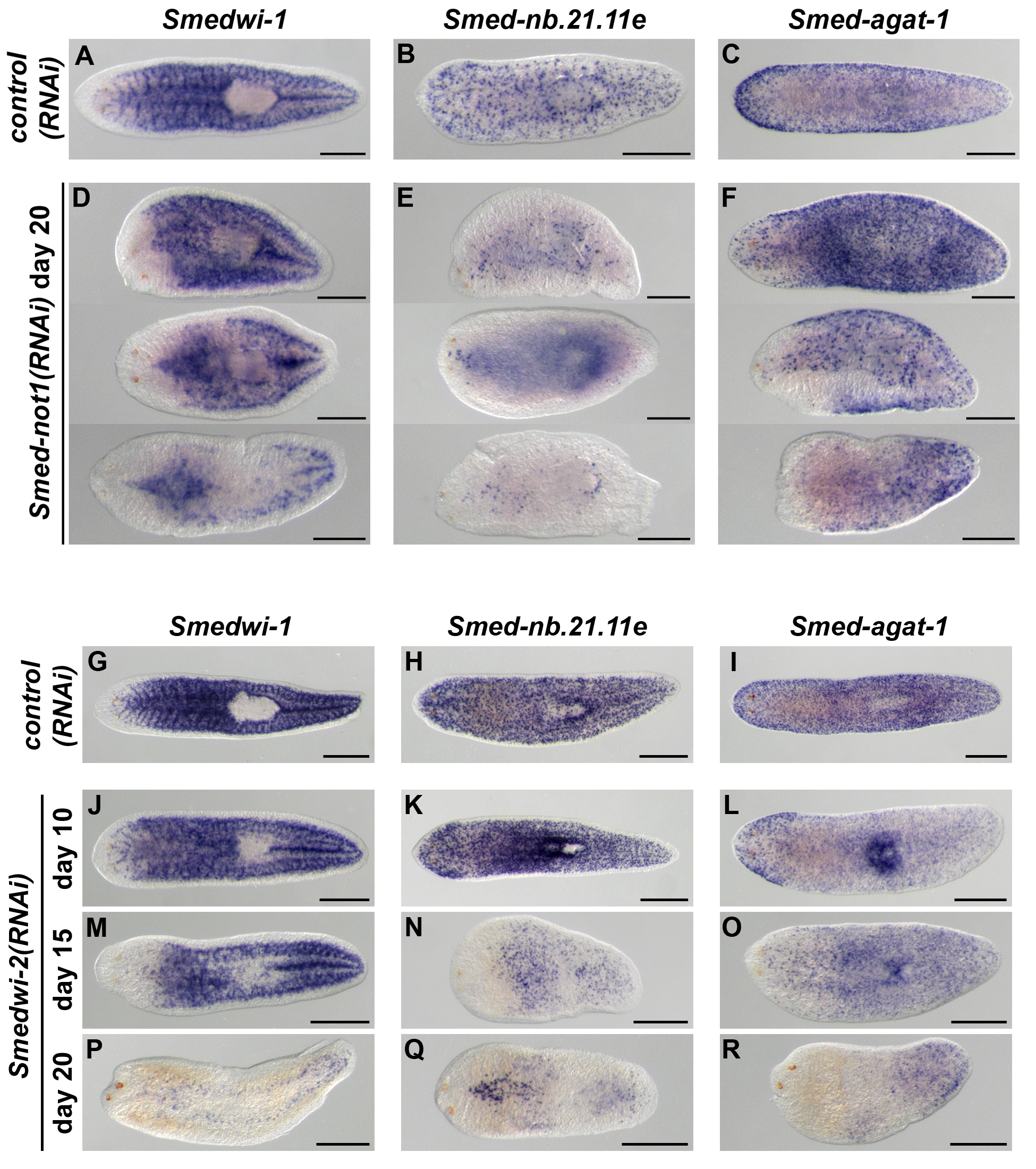

Supplement: Figure S5 — Dynamics of neoblasts and their progeny in Smed-not1(RNAi) and Smedwi-2(RNAi) animals. (A–F) WMISH of the neoblast marker Smedwi-1 (A, D), the early neoblast progeny marker Smed-nb.21.11e (B, E) and the late neoblast progeny marker Smed-agat-1 (C, F) in control(RNAi) animals (A–C) and Smed-not1(RNAi) animals (D–F) 20 days after RNAi. The level of Smedwi-1 signals in Smed-not1(RNAi) animals is variable, including animals with almost normal expression (D, top panel) and animals with a prominent reduction in Smedwi-1 levels (D, bottom panel). All Smed-not1(RNAi) animals present a severely reduced number of Smed-nb.21.11e-positive cells (E). The number of Smed-agat-1-positive cells is also variable (F), but all animals have reduced levels in the anterior part, typical behaviour of the marker Smed-agat-1 upon neoblast perturbation. (G–R) WMISH of the neoblast marker Smedwi-1 (G, J, M, P), the early neoblast progeny marker Smed-nb.21.11e (H, K, N, Q) and the late neoblast progeny marker Smed-agat-1 (I, L, O, R) in control(RNAi) animals (G–I) and Smedwi-2(RNAi) animals 10 (J–L), 15 (M–O) and 20 (P–R) days after RNAi. Smedwi-2(RNAi) animals have detectable expression of Smedwi-1 in almost all time points (J, M), although a severe decline in the level of Smedwi-1 signals is detected 20 days after RNAi (P). The dynamics of progeny markers is also abnormal, with a progressive decline of Smed-nb.21.11e signals (N, Q) and of Smed-agat-1 signals (L, O, R) that precedes the neoblast loss. Anterior is to the left. Scale bars: 500 µm. (TIF) [file pgen.1004003.s005.tif]

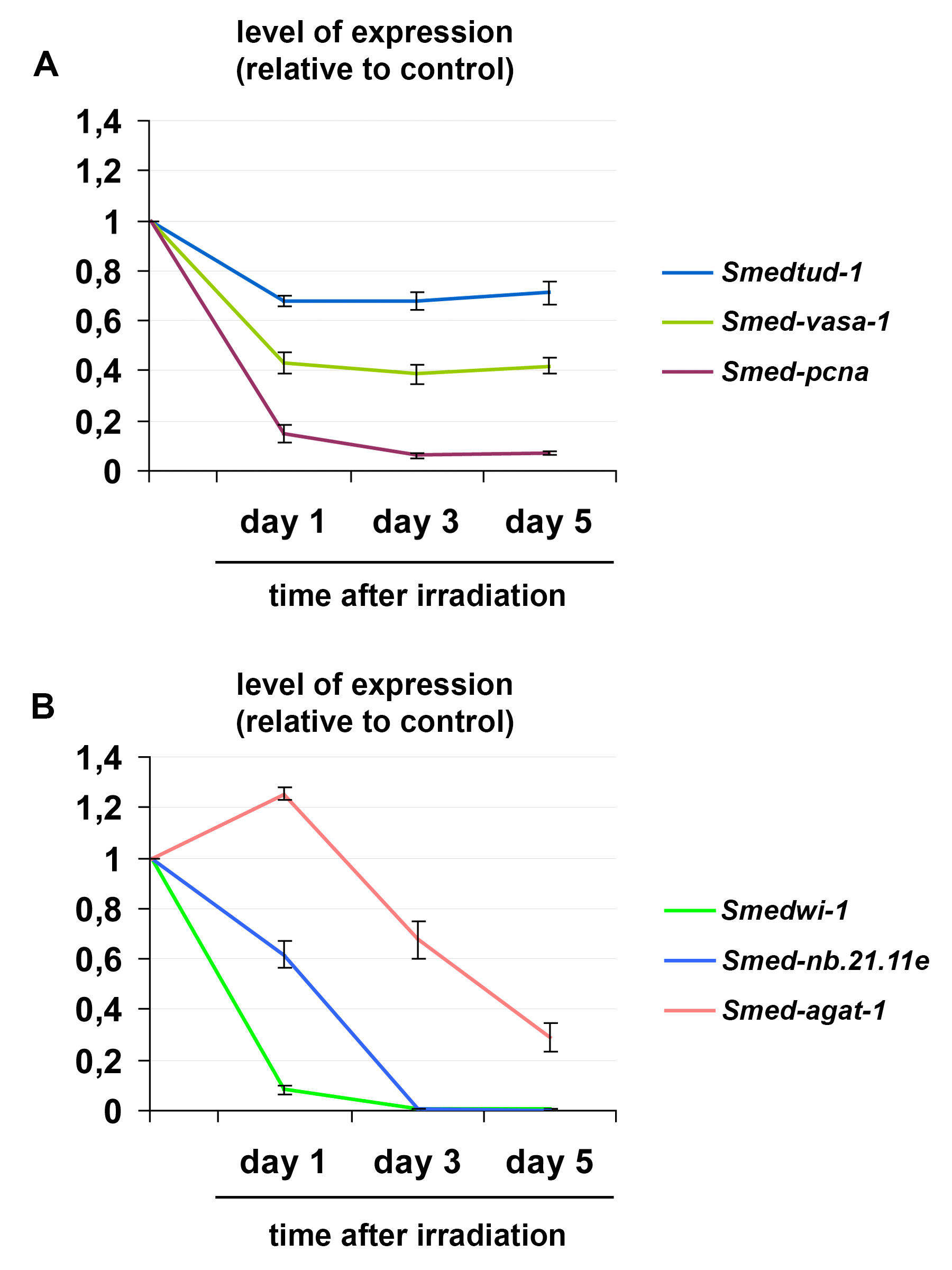

Supplement: Figure S6 — Dynamics of stem cell transcripts and progeny transcripts after irradiation. (A–B) Quantification of the level of expression by qRT-PCR of the stem cell markers Smedtud-1, Smedvas-1, and Smed-pcna (A) and of the neoblast and progeny markers Smedwi-1, Smed-nb.21.11e and Smed-agat-1 (B) in animals 1, 3 and 5 days after irradiation, normalized expression and relative to non irradiated samples. Error bars represent standard deviation. Animals 1 day after irradiation have around 10% of Smed-pcna transcripts of non-irradiated controls, and this number further decreases 3 and 5 days after irradiation. However, the expression of Smedtud-1 and Smedvas-1 mRNAs only decreases to around 70% and 40% respectively of the level of non irradiated controls, reflecting expression that does not localize to neoblasts and is therefore not eliminated by irradiation. Similar to Smed-pcna, the level of Smedwi-1 transcripts decreases to around 10% of the expression in non irradiated controls and becomes almost undetectable later. The levels of the progeny specific mRNAs Smed-nb.21.11e and Smed-agat-1 decrease progressively at later time points of irradiation. Therefore, around 90% of the neoblast specific transcripts are eliminated only 1 day after irradiation while most of the expression of progeny specific transcripts is still detected and the non-neoblast expression of Smedtud-1 and Smedvas-1 localized in the CNS is not eliminated by irradiation. (TIF) [file pgen.1004003.s006.tif]
