# Supplementary material for: The CCR4-NOT Complex Mediates Deadenylation and Degradation of Stem Cell mRNAs and Promotes Planarian Stem Cell Differentiation
Source: PLoS Genet. 2013 Dec 19;9(12):e1004003. doi: 10.1371/journal.pgen.1004003 (PMC3868585; doi:10.1371/journal.pgen.1004003)
Supplement: Table S1 — In silico search of CCR4-NOT complex components in S. mediterranea. Summary of the CCR4-NOT complex components found in silico in S. mediterranea. Each of the described components of the yeast, Drosophila melanogaster and human CCR4-NOT complexes is indicated. The column “S. mediterranea” indicates the given name for each of the genes, the column “Contig Smed genome” indicates the genomic contigs in which each locus was found, followed by the transcriptomic datasets from Blythe et al. and Adamidi et al. The column “D. japonica” indicates the accession numbers of the CCR4-NOT components previously described in this planarian species. Nine different components of the CCR4-NOT complex were found both in genomic and transcriptomic sequences, corresponding to the orthologues of all metazoan CCR4-NOT complex components. Similar to humans, two paralogues of the not7/caf1 gene were found (Smed-not7A and Smed-not7B) in both genomic and transcriptomic sequences and two additional genomic loci encoding two similar versions of an additional not7/caf1 gene were found (Smed-not7C.1 and Smed-not7C.2). However, the transcripts encoded by these two genomic loci were not found in transcriptomic datasets, and therefore they are possible pseudogenes. No orthologue of the yeast specific not5 was found, but one orthologue of the metazoan specific not10 was found (Smed-not10). We found several transcripts mapping to the same genomic locus for most of the genes, encoding different regions of the gene or different splicing variants. The Smed-not1 gene was split in two different contigs (v31.001778 and v31.002774) encoding respectively the 5′ and 3′ regions of the same gene. PCR experiments confirmed that they correspond to the same transcript. (PDF) [file pgen.1004003.s007.pdf]

name	yeast	*Drosophila*	Human	***S. mediterranea***	**Contig Smed genome**	**Transcriptome Blythe et al.**	**Transcriptome Adamidi et al.**		***D. japonica***
							**de novo**	**cufflinks**	
not1	not1p/cdc39	not1	cnot1	***Smed-not1***	v31.001778 - v31.002774	AAA.454ESTABI.5886	isotig18455	CUFF.103538.1	
						AAA.454ESTABI.12472		CUFF.103540.1	
						AAA.454ESTABI.16328		CUFF.132059.1	
not2	not2p/cdc36	regena	cnot2	***Smed-not2***	v31.001686	AAA.454ESTABI.4207	isotig16723		
not3	not3p	lethal (2) NC136	cnot3	***Smed-not3***	v31.007408	AAA.454ESTABI.5320	isotig24950		
						AAA.454ESTABI.23309			
not4	not4p/mot2p	CG31716	cnot4	***Smed-not4***	v31.000299	AAA.454ESTABI.23903	isotig13247		
						AAA.454ESTABI.23904	isotig13248		
not5	not5p	-	-	**-**	-	-	-		
not6/ccr4	ccr4p/fun27p	twin	cnot6-cnot6L	***Smed-not6***	v31.000014	AAA.454ESTABI.14907	isotig12027		GU305868
						AAA.454ESTABI.19158			
						AAA.454ESTABI.15937			
not7/caf1	caf1p/pop2p	pop2	cnot7-cnot8	***Smed-not7A***	v31.01247	AAA.454ESTABI.9392	isotig01978		GU305869
						AAA.454ESTABI.7911			
				Smed-not7B	v31.000784	AAA.454ESTABI.8927	isotig23596		
				Smed-not7C.1	v31.002031	-	-		
				Smed-not7C.2	v31.003641		-		
not9/caf40	caf40p	CG14213	rqcd1/cnot9	***Smed-not9***	v31.001835	AAA.454ESTABI.21764	isotig03690		
not10	-	CG18616	cnot10	***Smed-not10***	v31.000046	AAA.454ESTABI.10330	isotig14445		
